# Exploring the Utility of ALDH1 as a Marker for the Cancer Stem Cell Population in OCCC Cell Lines

**DOI:** 10.3390/cells15171509

**Published:** 2026-08-22

**Authors:** Blane Gebreyes, Bart Kolendowski, Yudith Ramos-Valdes, Trevor G. Shepherd, Gabriel E. DiMattia

**Affiliations:** 1 Mary & John Knight Translational Ovarian Cancer Research Unit, Verspeeten Family Cancer Center, London, ON N6A 4L6, Canada; bgebreye@uwo.ca (B.G.); bkolendo@uwo.ca (B.K.); yudith.ramos-valdes@lhsc.on.ca (Y.R.-V.); trevor.shepherd@schulich.uwo.ca (T.G.S.); 2 Department of Biochemistry, Western University, London, ON N6A 3K7, Canada; 3 London Health Sciences Centre Research Institute, London, ON N6A 4L6, Canada; 4 Department of Oncology, Western University, London, ON N6A 3K7, Canada; 5 Department of Anatomy & Cell Biology, Western University, London, ON N6A 3K7, Canada; 6 Department of Obstetrics & Gynaecology, Western University, London, ON N6A 3K7, Canada

**Keywords:** ALDH1A1, cancer stem cells, ovarian clear cell carcinoma, spheroids, metastasis, chemoresistance

## Abstract

Metastasis, chemoresistance, and tumour recurrence are facilitated by cancer stem cells (CSCs), a small subpopulation of cells capable of regenerating a primary tumour while maintaining the tumour’s genetic and phenotypic features. CSCs can be identified by the expression of specific markers; however, the CSC population in ovarian clear cell carcinoma (OCCC), a rare histotype of ovarian cancer, remains poorly defined. Given the well-established role that CSCs play in cancer progression and metastasis, it is critical to identify reliable markers of CSCs in OCCC. Here, we endeavoured to determine whether ALDH1 expression could be used to define OCCC stem cells in OCCC cell lines using a variety of methods including assessing ALDH1A1 expression in spheroids generated under distinct conditions. We also generated and used chemo-resistant cell lines to assess the enrichment of cancer stem cells. Human OCCC cell lines were enriched for CSCs using selective culture conditions and drug resistance methods. CSC-enriched spheroids demonstrated increased expression of stemness markers NANOG and SOX2, while ALDH1A1 expression was enriched only in drug-resistant cell lines, relative to parental cell lines. RNA-seq analyses of CSC-media-derived spheroids versus standard media spheroids provided novel data supporting CSC enrichment and identified transcription factors induced by CSC media. These findings highlight the ambiguous role of ALDH1A1 as a CSC marker in OCCC and demonstrates the utility of CSC enrichment methods for identifying CSC populations in OCCC cell lines.

## 1. Introduction

Epithelial ovarian cancer (EOC) is the most lethal gynecological cancer in the developed world and is projected to be the fifth most deadly cancer among women in 2026 [1]. Like other cancers, early diagnosis and intervention of EOC can increase survival rates; however, even with early detection, EOC remains one of the leading causes of cancer-related deaths. A key contributor to EOC mortality is the high rate of metastasis at the time of diagnosis, occurring in 70–75% of patients. This is in large part facilitated by the passive exfoliation of cells off the primary tumour, which are then able to form multicellular aggregates, called spheroids, that spread through the peritoneal cavity [2,3].

Ovarian clear cell carcinoma (OCCC) is a rare subtype of EOC, making up 5–10% of cases in North America [4]. OCCC is distinct from other EOCs as it originates from endometrial tissue that underwent retrograde transport from the uterus to the surface of the ovaries or other peritoneal surfaces in a process associated with endometriosis, rather than being derived from ovarian tissue [5,6,7,8,9]. The molecular profile of OCCC also differs from other EOCs, with tumours commonly displaying mutations in ARID1A (inactivation in ~50% of cases), PI3KCA (hyperactivation, ~40%), and MET (amplification, ~37%). OCCC tumours also display low level of TP53 mutation, contrasting with high-grade serous ovarian carcinoma (HGSOC), the most prevalent subtype of EOC, where TP53 is ubiquitously mutated [4,10,11,12].

OCCC has been shown to be less responsive to the pan-EOC standard-of-care chemotherapeutics paclitaxel and carbo- or cisplatin, with initial chemotherapy response rates of 10–25%. This is contrasted by other EOC subtypes, like HGSOC and endometrioid carcinoma, which display response rates closer to 80% [4,13,14,15,16]. Despite the dissimilarity of OCCC tumours to other EOCs, paclitaxel and platinum-based therapies remain the standard-of-care chemotherapeutics, as no effective therapeutic interventions have been identified for OCCC [17,18].

Metastasis and chemoresistance can be attributed, in part, to the presence of cancer stem cells (CSCs), a malignant subpopulation of cells thought to make up less than 1% of the average tumour mass. This subpopulation is defined by their unique properties of self-renewal and multipotency [19,20]. CSCs share several traits with normal stem cells and can be identified by a unique profile of cell surface and intracellular markers in both hematological and solid tumours [20,21,22,23,24,25]. Within EOC, CSCs have primarily been characterized in HGSOC and have largely involved markers such as CD24, CD44, CD117, CD133, and ALDH1A1 as those which define, identify, or are implicated in HGSOC CSCs [26,27,28,29,30,31,32]. However, the association of HGSOC CSC markers in the OCCC histotype is not understood.

One of these markers, aldehyde dehydrogenase 1A1 (ALDH1A1), is one of the most widely implicated indicators of stemness in both normal stem cells and CSCs of several cancer types, such as breast, prostate, esophageal, and HGSOC CSCs [29,33,34,35,36]. ALDH1A1 is responsible for the oxidation of retinaldehyde (retinal) to retinoic acid, a process that is essential for cell growth, differentiation, and development, and plays a critical role in maintaining stem cell characteristics such as resistance to exogenous compounds and regeneration [37,38]. The Yamanaka factors, OCT4 (POU5F1), SOX2, KLF4, and c-MYC, are a set of transcription factors implicated in reprogramming differentiated cells to become iPSCs with traits similar to embryonic stem cells [39,40]. The expression of these factors, along with the transcription factor NANOG, can also be seen in CSCs, allowing CSCs to exhibit similar stem-like traits as those seen in iPSCs [39]. In CSCs, these factors are responsible for maintaining the stem-like population, promoting tumour invasiveness, and chemoresistance [41,42,43,44,45].

Given the unique treatment response, etiology, and molecular characteristics of OCCC, uncovering the relevance of ALDH1A1 expression in defining OCCC CSCs is central to our understanding of OCCC-CSC mediated metastasis and treatment resistance. Furthermore, defining the molecular features of human OCCC CSCs is foundational to uncovering biochemical vulnerabilities which could be used to specifically eliminate these cells, reducing tumour burden and preventing treatment resistance and disease recurrence.

Here, we have focussed on ALDH1A1 and the properties of enriched CSCs using established human OCCC cell lines. We hypothesize that human OCCC cell lines carry a subpopulation of cells which exhibit CSC characteristics based on the expression and activity of ALDH1A1. We have incorporated a large number of established human OCCC cell lines, including novel paclitaxel and AZD-8055-resistant cell lines, to identify lines which may be particularly valuable in the characterization of OCCC CSCs. Our analysis highlights the ambiguous role played by ALDH1A1 in OCCC stem-like cells, positioning its expression to be more closely associated with supporting stemness in response to therapy resistance rather than being a generalizable marker of OCCC CSCs.

Using bulk RNA-seq, we further explored the transcriptional basis for CSC-associated growth advantages in spheroid culture, and the effects of paclitaxel resistance on stemness. Interestingly we discovered a significant change in the transcriptome of the OCCC spheroids in CSC media after 7 days of culture when compared to standard media-derived spheroid cells. Moreover, we compared the transcriptomes of a paclitaxel-resistant cell line under these conditions to its parental line to determine whether CSCs were enriched by chemoresistance. Ultimately, while CSC spheres displayed molecular characteristics found in CSCs of other cancers and normal stems cells, and were enriched with stem cell media, we found that ALDH1A1 is not strictly associated with the CSC phenotype from established OCCC cell lines. However, we identified transcriptional regulators such as SOX9, ETV4, and ETV5 as promising candidate markers for further investigation. The RNA-seq data are expected to provide a platform with which to identify biochemical pathways amenable to therapeutic targeting to eradicate CSCs in OCCC.

## 2. Materials and Methods

### 2.1. Cell Culture

The human OCCC cell lines 105C, TOV-21G, KOC-7c, RMG-V, SMOV-2, OVTOKO, OVMANA, TU-OC-1, RMG-II, RMG-I, JHOC-5, OV207, and ES-2 cell lines have been previously described [46,47]. The 12Z cell line is an SV40 T-antigen immortalized human endometrial epithelial cell line and was obtained from Dr. Ronald Chandler (Michigan State University, East Lansing, MI, USA). The EFO-21 and EFO-27 cell lines were purchased from the Leibniz Institute DSMZ, German Collection of Microorganisms and Cell Cultures GmbH (Braunschweig, Germany). Cell lines were authenticated using short tandem repeat (STR) analysis performed at the Centre for Applied Genomics (TCAG) (The Hospital for Sick Children, Toronto, ON, Canada) and confirmed negative for mycoplasma (ATCC, 30-1012K). For clarity purposes, please see Supplementary Table S1 for a listing of the cell lines used in this study and which assays were applied to each line.

All cell lines were maintained in adherent culture in Dulbecco’s Modified Eagle Medium/Ham’s F12 (DMEM/F12) (Gibco, Grand Island, NY, USA, catalogue #11330057), supplemented with 10% fetal bovine serum (FBS) (Wisent Inc., Saint-Jean-Baptiste, QC, Canada, catalogue #098-150-CL), and were grown in a humidified incubator at 37 °C with 95% ambient air and 5% CO_2_. Cells were maintained in 10 cm tissue culture-treated polystyrene plates (Sarstedt, Newton, NC, USA, catalogue #83.3902) and passaging was performed at 80–90% confluency. Cells were washed once with Dulbecco’s Phosphate-Buffered Saline (PBS) (Wisent Inc., catalogue #311-425-CL) and treated 0.25% trypsin/0.1% EDTA (Wisent Inc., catalogue #325-045-EL) to obtain a single cell suspension. Trypsin/EDTA was neutralized with complete media, and cells were replated at a 1:2–1:10 dilution.

Spheroids were generated and cultured in either DMEM/F12, supplemented with 10% FBS, denoted henceforth as standard (STD) media, or Advanced DMEM/F12 (Gibco, catalogue #12634028), supplemented with 10 mM HEPES (Wisent Inc., catalogue #330-050-EL), 1× Insulin-Transferrin-Sodium Selenite Supplement (ITSS) (Roche, Mississauga, ON, Canada, catalogue #11074547001), 40 ng/mL Heparin (Sigma-Aldrich, Burlington, MA, USA, catalogue #H3149), 40 ng/mL bFGF (Novus Biologicals, Toronto, ON, Canada, catalogue #NBP2-34921-100), 20 ng/mL hEGF (PeproTech, Cranbury, NJ, USA, catalogue #AF-100-15-500UG), 1× B27 (Gibco, Mississauga, ON, Canada, catalogue #17504044), and 1× Glutamax (Gibco, catalogue #35050061), denoted henceforth as cancer stem cell (CSC) media [48,49]. Spheroids formed autonomously by culturing cells on ultra-low attachment (ULA) plates (Corning, Corning, NY, USA, catalogue #3471, 3473, 3474). Spheroids in both media compositions were cultured in a humidified incubator at 37 °C with 95% ambient air and 5% CO_2_.

Prior to seeding spheroids in STD and CSC media, adherent cells in serum-containing media were washed once with PBS. Cells were treated with TrypLE (Gibco, catalogue #12605010) and incubated in a humidified incubator at 37 °C with 95% ambient air and 5% CO_2_ to obtain a single cell suspension. After incubation, TrypLE was neutralized using PBS. Cells were pelleted at 1000× *g* and resuspended in fresh PBS prior to counting with 0.4% Trypan Blue Stain (Gibco, catalogue #15250061; Invitrogen, Waltham, MA, USA, catalogue #T10282).

### 2.2. Reverse Transcription Quantitative Polymerase Chain Reaction (RT-qPCR)

Adherent cells were seeded at a density of 0.5–1 × 10^6^ cells per 10 cm plate and cultured in a humidified incubator at 37 °C with 95% ambient air and 5% CO_2_ for 3 days prior to RNA lysate preparation. Cells were washed twice with cold PBS, then gently scraped in RLT buffer from the RNeasy Mini Kit (Qiagen, Hilden, Germany, catalogue #74106), supplemented with 1% *v*/*v* β-mercaptoethanol (BME). RNA was isolated according to the manufacturer’s RNeasy Mini Kit protocol, including the intermediate DNase step using the RNase-Free DNase Set (Qiagen, catalogue #79256). Concentration and quality were measured using the Thermo Scientific (Mississauga, ON, Canada) NanoDrop One Microvolume UV-Vis spectrophotometer.

Reverse transcription was performed using the High-Capacity cDNA Reverse Transcription Kit (Applied Biosystems, Waltham, MA, USA, catalogue #4368814) according to the manufacturer’s protocol. Reverse transcription included “no reverse transcriptase” control reactions to ensure no genomic DNA contamination in isolated RNA. End-point PCR (Promega, Madison, WI, USA, catalogue #M791A; Promega, catalogue #M008; Invitrogen, catalogue #10297018) using human-specific GAPDH primers was performed using the Bio-Rad (Hercules, CA, USA) MyCycler thermal cycler with cDNA generated with and without reverse transcriptase, and amplicons were run on a 1% *w*/*v* agarose gel to ensure no genomic DNA contamination. Quantitative PCR was performed using the Brilliant III Ultra-Fast SYBR Green qPCR Master Mix (Agilent Technologies, Mississauga, ON, Canada, catalogue #600882) and the PerfectStart^®^ Universal Green qPCR SuperMix (GeneBio Systems Inc., Burlington, ON, Canada, catalogue #AQ602-02) using the Applied Biosystems (Waltham, MA, USA) QuantStudio3 Real-Time PCR System according to the manufacturer’s protocol. Ct values were analyzed using the ΔΔCt method relative to the 105C cell line using Microsoft Excel (Microsoft 365 Version 16.112.1, 81 Bay St. Toronto, ON, Canada). All DNA primers used for RT-qPCR along with source of the primers are listed in Table 1 below.

### 2.3. Immunoblotting

All antibodies used in this work along with commercial sources and dilutions used for Western blotting are listed in Table 2 below. Adherent cells were seeded at a density of 0.5–1 × 10^6^ cells per 10 cm plate and cultured in a humidified incubator at 37 °C with 95% ambient air and 5% CO_2_ for 3 days prior to whole cell lysate preparation. Spheroids were seeded at a density of 1–2 × 10^5^ cells/well in 6-well ULA plates (Corning, catalogue #3471) and cultured for 3 or 7 days in a humidified incubator at 37 °C with 95% ambient air and 5% CO_2_ prior to whole cell lysate preparation. Adherent cells were washed twice with cold PBS, then gently scraped in radioimmunoprecipitation assay lysis buffer (RIPA; 50 mM Tris HCl, 150 mM NaCl, 1.0% *v*/*v* IGEPAL CA-630, 0.5% *w*/*v* Sodium Deoxycholate, 1.0 mM EDTA, 0.1% *w*/*v* SDS, 0.01% *w*/*v* sodium azide pH 7.4) supplemented with 225 mM sodium pyrophosphate, 0.5 M Sodium Fluoride, 200 mM Sodium Orthovanadate, 10× protease inhibitor cocktail (Sigma-Aldrich, catalogue # S8820-2TAB), 1 mM PMSF, and 10 mM β-Glycerophosphate. For spheroids, cells were harvested from ULA plates and centrifuged at 1000× *g* for 5 min. The supernatant was discarded and cell pellets were washed twice with cold PBS, after which they were lysed with RIPA buffer containing the aforementioned supplements. The resulting cell lysate mixtures were further lysed by brief sonication. Lysates were then centrifuged at 14,000× *g* for 30 min at 4 °C. The supernatant was isolated, and protein concentration was quantified using the Bradford assay with Protein Dye Reagent (Bio-Rad, Hercules, CA, USA, catalogue #500-0006).

Protein samples were prepared so that samples contained 20 µg protein, and electrophoretic separation was performed using the Bio-Rad Mini-PROTEAN II Electrophoresis System following the manufacturer’s instructions using 8–14% SDS-polyacrylamide gels. Proteins were transferred to polyvinylidene fluoride (PVDF) membranes (Thermo Scientific, Mississauga, ON, Canada, catalogue # 88518) and subsequently blocked for 1 h with either 5% *w*/*v* skimmed milk or 5% *w*/*v* bovine serum albumin (BSA) in Tris-buffered saline with Tween 20 (TBST; 20 mM Tris, 150 mM NaCl, and 0.1% Tween 20), based on primary-antibody-specific manufacturer instructions. Primary antibodies were diluted according to the manufacturer’s recommendations and incubated with the membranes overnight at 4 °C. After overnight incubation, membranes were washed 3 × 5 min with TBST, then probed with either mouse or rabbit horseradish peroxidase (HRP)-conjugated secondary antibodies for 1 h at room temperature. Following secondary antibody incubation, membranes were washed 3 × 5 min with TBST. Enhanced chemiluminescence (ECL) detection was achieved by incubating membranes in Immobilon Forte Western HRP Substrate (MilliporeSigma, Oakville, ON, Canada, catalogue #WBLUF0500) for 3–5 min, based on protein abundance. Western blot images were acquired using the Bio-Rad ChemiDoc Imaging System, Image lab Touch Software version 3.0.1.14 (1329 Meyerside Drive, Mississauga, ON, Canada). Western blot images have been cropped for presentation.

### 2.4. Flow Cytometry

Cells were seeded at a density of 0.5–3 × 10^6^ cells per 10 cm plate and were allowed to grow in adherent culture for 3 days. After 3 days, cells were harvested and pelleted at 1000× *g* for 5 min. Pellets were resuspended in ALDEFLUOR^TM^ buffer from the ALDEFLUOR^TM^ kit (STEMCELL Technologies Canada Inc., Vancouver, BC, Canada, catalogue #01700) at a density of 2 × 10^6^ cells/mL. Approximately 1 × 10^6^ cells were combined with BODIPY-aminoacetaldehyde (BAAA) and mixed well, immediately after which approximately 5 × 10^5^ cells were transferred to a separate tube and co-incubated with the specific ALDH inhibitor 4-diethylaminobenzaldehyde (DEAB) for 45 min at 37 °C in the dark. After incubation, all samples were pelleted at 300× *g* at 4 °C for 5 min and washed twice with ALDEFLUOR^TM^ buffer. 7-aminoactinomycin D (7AAD) (BD Bioscience, Mississauga, ON, Canada, catalogue #559925) viability dye was added to each sample, and samples were incubated at RT for 5 min. Single stain control tubes were similarly prepared for each fluorescent target assessed. Samples were analyzed on the Beckman Coulter (Mississauga, ON, Canada) DxFlex flow cytometer, and the resulting data were analyzed using FlowJo (BD Life Sciences, 2100 Derry Road West, Suite 100, Mississauga, ON, Canada) v10.10.1.

### 2.5. Determination of Half-Maximal Inhibitory Drug Concentration

Adherent cells were seeded at 1.5–3 × 10^3^ cells/well in 96-well adherent plates (Sarstedt, catalogue #83.3924). After overnight attachment, cells were treated with a 12-point, 2-fold serial dilution of paclitaxel (Caymen Chemicals, Ann Arbor, MI, USA, catalogue #10461), beginning at the following concentrations: OVTOKO: 16 µM; TOV-21G: 100 nM; 105C: 200 nM; or AZD-8055 (MedChemExpress, Monmouth Junction, NJ, USA, catalogue #HY-10422) for 105C only, beginning at 12.8 µM, in triplicate wells. Dimethyl sulfoxide (DMSO)-treated cells were used as a vehicle control for both drugs, treated at a concentration equal the highest concentration of paclitaxel or AZD-8055 for each respective cell line. After 3 days of treatment, alamarBlue^TM^ cell viability reagent (Invitrogen, catalogue #DAL1100) was used to quantify treatment efficacy on cell viability, and fluorescence (555, 596 nm) was measured after 4 h of incubation using the Agilent (Mississauga, ON, Canada) BioTek Synergy H1 plate reader. Absorbance measurements for the lowest drug concentration were used to establish a baseline of 100% viability, then viability measurements for each concentration were set as a percentage relative to the baseline. GraphPad Prism (GraphPad Software) v10.4.1 was used to calculate IC50 concentrations by fitting a curve to the baseline-corrected data using non-linear regression.

### 2.6. Development of Drug-Resistant OCCC Cell Lines

Adherent cells were plated at a density of 2.5–5 × 10^5^ cells per 10 cm plate and were exposed to increasing concentrations of paclitaxel or AZD-8055, starting at 5% of the IC50, followed by concentrations of drug that increased by 10% of the IC50 at each subsequent passage. Resistant cells were exposed to an elevated concentration of drug at the time of passaging if cell viability was above 75%, as determined using the Bio-Rad TC10 Automated Cell Counter; otherwise, cells were passaged at the same concentration until a suitable tolerance was achieved. Once a satisfactory level of resistance was achieved, cells were maintained in their last dose indefinitely (Table 3). Cells are never removed from maintenance concentrations of drug except for lysate generation or specific experiments, unless otherwise stated.

### 2.7. OCCC Cell Line Spheroid Screen

Cells were seeded into 96-well flat-bottom ULA plates (Corning, catalogue #3474) at densities ranging from 250 to 1500 cells/well in CSC media, in triplicate wells. Spheroid development was monitored via imaging using the Sartorius IncuCyte S3 (Sartorius, Oakville, ON, Canada) over a period of 10 days.

### 2.8. Trypan Blue Exclusion Cell Counting of Spheroids

Cells were seeded at 1.0 × 10^4^ cells/well in 24-well ULA plates (Corning, catalogue #3473) in either STD or CSC media in triplicate wells. For MK-1775 (Caymen Chemicals, catalogue #21266) treated spheroids, media containing the listed concentration of drug, along with media containing DMSO at a concentration equal to the highest concentration of MK-1775, was added after 3 days in suspension culture for 4 days, resulting in a total time in suspension of 7 days. For resistant spheroids, cells were treated at maintenance concentrations of paclitaxel or AZD-8055 at the time of seeding (Table 3). After 7 days, spheroids in triplicate wells were combined into one microcentrifuge tube to ensure the total cell number was within the counting range of the automated cell counter. Spheres were pelleted at 1500× *g*, then washed with PBS. After pelleting again, residual PBS was thoroughly removed, and spheroids were resuspended in 25 μL TrypLE and incubated in a 37 °C water bath for 30 min to obtain a single cell suspension. TrypLE was neutralized with 5 μL 50% FBS in PBS. Additional PBS was added to samples predicted to have high cell numbers. Trypan blue stain was added at a volume equivalent to the total volume of the cell suspension, and samples were counted 4 times using the Invitrogen (Waltham, MA, USA) Countess 3 Automated Cell Counter or Bio-Rad TC10 Automated Cell Counter, as indicated. Total cell count was divided by 3 to obtain the average cell number per well.

### 2.9. Spheroid Reattachment

Cells were seeded at 1.0 × 10^4^ cells/well in 24-well ULA plates in either STD or CSC media in triplicate wells and allowed to grow for 7 days. After 7 days, spheroids were transferred along with media, well for well, into 24-well adherent culture plates (Sarstedt, catalogue #83.3922) and then supplemented with an additional 0.5 mL of STD media. Spheroids were allowed to attach for 3 days. Once attached, cell viability was quantified using alamarBlue^TM^ cell viability reagent, and fluorescence (555, 596 nm) was measured after 4 h of incubation using the Agilent BioTek Synergy H1 plate reader.

Reattached cell biomass was quantified using crystal violet. AlamarBlue-containing media was removed, and spheres were fixed using cold 25% *v*/*v* methanol in PBS. Fixative was removed, and attached spheres were stained using 0.5% *w*/*v* crystal violet in 25% *v*/*v* methanol and PBS staining solution for 1 h, with rocking. The staining solution was removed from the wells, and the plate was carefully washed with water. The plate was left upside down to dry overnight. The following day, crystal violet was extracted using 10% *v*/*v* acetic acid in ddH2O for 1 h with shaking at 100 rpm using the Fisher Scientific (Ottawa, ON, Canada) Clinical Rotator. Eluted dye was collected into a microcentrifuge tube, well for well, and absorbance (590 nm) was measured using the Agilent BioTek Synergy H1 plate reader (6705 Millcreek Dr., Unit 5, Mississauga, ON, Canada).

### 2.10. RNA-Seq Analyses of OCCC Spheroid Transcriptomes

RNA-seq sample preparation and analysis for this work are as previously described [46]. Spheroids were seeded at a density of 1–2 × 10^5^ cells/well in 6-well ULA plates and cultured for 7 days in a humidified incubator at 37 °C with 95% ambient air and 5% CO_2_. To isolate RNA, spheroids were harvested from ULA plates and centrifuged at 1000× *g* for 5 min. The supernatant was discarded and cell pellets were washed twice with cold PBS after which they were lysed with RLT buffer from the RNeasy Mini Kit (Qiagen, catalogue #74106), supplemented with 1% *v*/*v* β-ME. RNA was isolated according to the manufacturer’s RNeasy Mini Kit protocol, including the intermediate DNase step using the RNase-Free DNase Set (Qiagen, catalogue #79256). Concentration and quality were measured using 5K/RNA/Charge Variant Assay LabChip and RNA Assay Reagent Kit (Perkin Elmer, Waltham, MA, USA). Libraries were generated from 250 ng of total RNA as following: mRNA enrichment and library preparation were performed using the Illumina Stranded mRNA Prep Kit (Illumina, San Diego, CA, USA), as per the manufacturer’s recommendations. Libraries were quantified using the KAPA Library Quantification Kits -Complete kit (Universal) (Kapa Biosystems, Wilmington, MA, USA). Average fragment size was determined using a Fragment Analyzer 5300 (Agilent, Santa Clara, CA, USA) instrument. The libraries were normalized and pooled and then denatured in 0.02 N NaOH and neutralized using pre-load buffer. The pool was loaded at 150 pM on an Illumina NovaSeq X Plus 25B lane following the manufacturer’s recommendations. The run was performed for 2 × 100 cycles (paired-end mode). A phiX library was used as a control and mixed with libraries at 1% level. Program BCL Convert 4.2.4 was then used to demultiplex samples and generate fastq reads. Initial processing was performed on the Galaxy platform: Raw reads were assessed with FastQC (v. 0.12.1), aligned to the human genome build hg38 using HISAT2 (v. 2.2.1) (default settings), and quantified with featureCounts. Differential expression was then determined using limma-voom (v. 3.58.1) using TMM normalization. Low count genes were filtered out (CPM ≤ 0.5 in greater than 2 samples were excluded).

Expression heatmaps, log-fold-change scatterplots, and set-intersection (UpSet) plots were generated in Python (v3.12.13) using pandas (v3.0.3), NumPy (v2.4.6), and matplotlib (v3.10.9). Gene-level differential-expression statistics (log2 fold changes, moderated t-statistics, and Benjamini–Hochberg adjusted *p*-values) were obtained from limma-voom analysis in Galaxy (v26.1), using limma (v3.58.1) and edgeR (v4.0.2) under R (v4.3.2), on reads aligned with HISAT2 (v2.2.1) and quantified with featureCounts (v 2.1.1). Scatterplots compared per-gene log fold changes between paired contrasts, and UpSet plots summarized the overlap of significantly differentially expressed genes (adjusted *p* < 0.05) across cell lines. Cell-type over-representation analysis was performed using Enrichr (https://maayanlab.cloud/Enrichr/, accessed on 1 June 2026). All figures were produced by custom scripts directly from the differential-expression and normalized-count tables.

### 2.11. Statistics

Statistical analysis was performed using GraphPad Prism v10.4.1. Specific analysis details are described in the figure legends.

## 3. Results

### 3.1. OCCC Cell Lines Demonstrate a Wide Range of ALDH1A1 Expression and Activity

**Figure 1:**
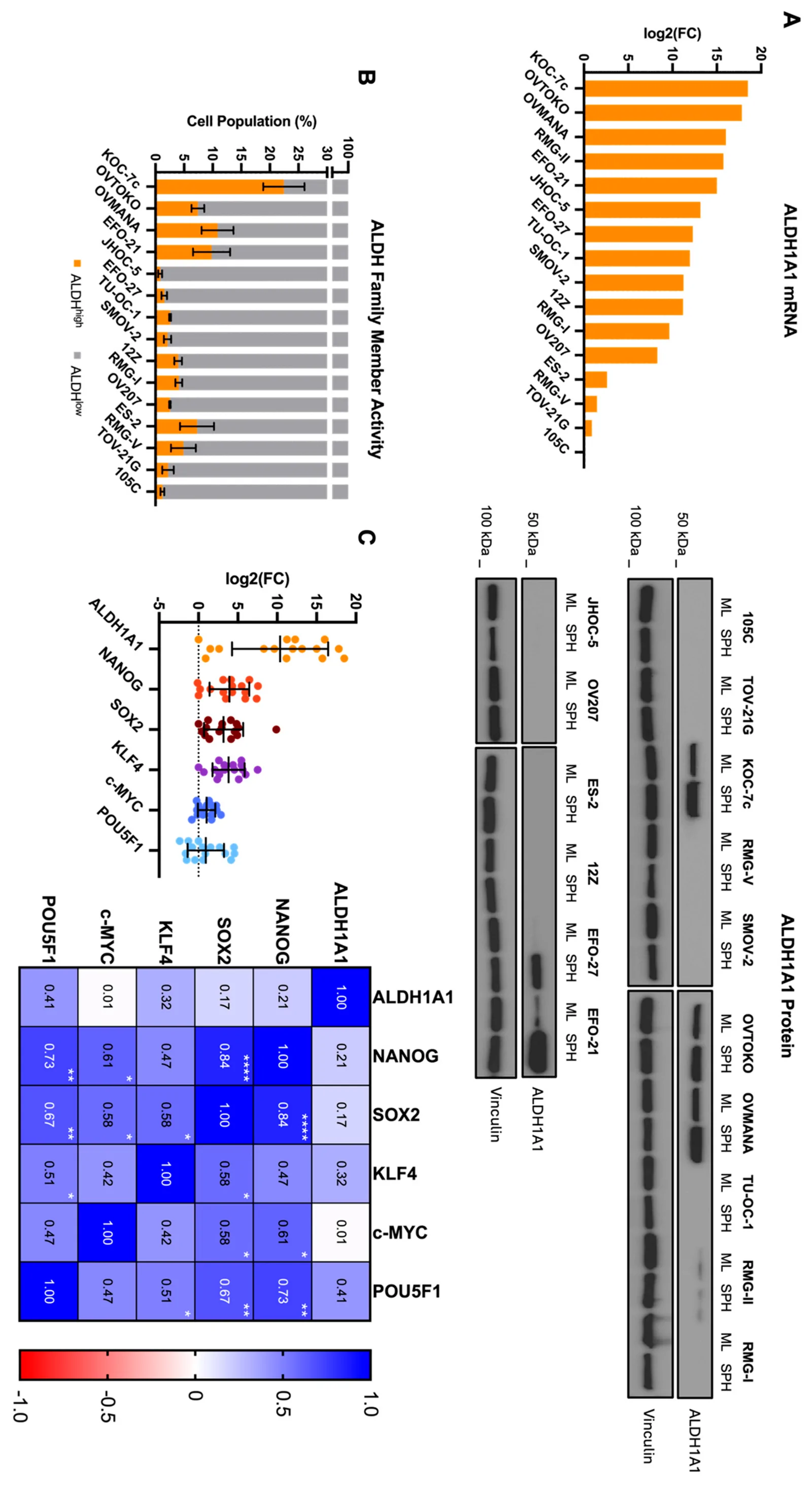
Assessment of stem cell marker expression in OCCC cell lines. (**A**) ALDH1A1 mRNA expression in adherent cells, determined by RT-qPCR, and protein expression of ALDH1A1 in adherent (ML) and spheroid (SPH) cells, determined by Western blot, in 16 OCCC cell lines cultured in standard media. (**B**) Survey of aldehyde dehydrogenase (ALDH) protein family activity using flow cytometry from 15 different OCCC cell lines. Data represent 3 independent biological replicates. (**C**) Log-transformed expression of stem cell markers NANOG, SOX2, KLF4, c-MYC, and POU5F1, determined by RT-qPCR, relative to the lowest *ALDH1A1*-expressing cell line, 105C. Spearman correlation matrix of mRNA expression, determined by RT-qPCR, between *ALDH1A1*, *NANOG*, *SOX2*, *KLF4*, *c-MYC*, and *POU5F1* across 16 OCCC cell lines; * *p* < 0.05, ** *p* < 0.01, **** *p* < 0.0001. Data represent 16 independent biological replicates.

An initial analysis of CSC markers, chosen based on the literature, was performed at the mRNA and protein level, using established OCCC cell lines cultured in standard media. Screening several different established OCCC cell lines allowed for a survey of potential baseline stem-like characteristics prior to functional screening. We assessed levels of ALDH1A1 mRNA and protein in adherent and spheroid culture and found a wide range of expression levels in OCCC cell lines (Figure 1A, Supplementary Figure S1). Generally, lines with high ALDH1A1 mRNA levels, such as KOC-7c, OVTOKO, and OVMANA, also showed high protein levels, that increased in STD media (DMEM/F12, 10% FBS) spheroid culture relative to adherent culture. This is contrasted by RMG-II cells, which showed high *ALDH1A1* mRNA expression, but low protein levels in both adherent and spheroid culture. EFO-21 and EFO-27 exhibited the largest increase in ALDH1A1 protein expression from adherent to spheroid culture. It should be noted that the mRNA abundance data are plotted as the log2-transformed fold changes relative to the 105C cell line which is the lowest *ALDH1A1*-expressing OCCC cell line. Hence, the linear fold-change values were consistent with the Western blot data, where only cell lines with detectable ALDH1A1 protein also showed high levels of *ALDH1A1* mRNA.

Lines with high *ALDH1A1* mRNA and protein also demonstrated a large proportion of cells exhibiting high ALDH activity, as demonstrated by the flow cytometric ALDEFLUOR^TM^ assay (Figure 1B). This is consistent with mRNA and protein levels of ALDH1A1 as OCCC lines that displayed high expression also showed high ALDH family member activity.

Next, mRNA levels of *NANOG*, *SOX2*, *KLF4*, *c-MYC*, and *POU5F1* were assessed in adherent cells cultured in STD media to obtain baseline data. Interestingly, while there was a wide range of expression levels of ALDH1A1, there was a narrower range in expression for the other markers. A Spearman correlation analysis was performed to determine whether there was a correlation between the mRNA expression of *ALDH1A1* and that of *NANOG*, *SOX2*, *KLF4*, *c-MYC*, and *POU5F1*. Interestingly, this correlation analysis demonstrated a low correlation between ALDH1A1, and the expression of the other CSC-associated markers, with only a moderate non-significant correlation with *KLF4* expression across all OCCC cell lines (Figure 1C). The other markers, assessed at the mRNA level, displayed significant moderate to strong correlations between each other, barring that between *KLF4* with *NANOG* and *c-MYC*, and *POU5F1* with *c-MYC*, whose correlations only approached significance (Supplementary Table S2). This correlative relationship is consistent with the cooperative regulatory dynamic between these marks in maintaining self-renewal in both regular stem cells and CSCs [43,44,45]. Overall, expression of the examined CSC markers is highly variable across OCCC cell lines, indicating that marker expression patterns are cell-line-dependent within a general bulk population of cells. An overview of these results is presented in Supplementary Table S3.

### 3.2. OCCC Spheroids Demonstrate Viability and Proliferation in CSC Media

A functional screen was performed to qualitatively assess cell viability and proliferation in CSC media suspension culture between various OCCC cell lines. This was to assign a functional output to bulk cell marker expression, especially as marker expression was varied across the lines examined. A large number of OCCC cell lines were initially assessed to determine which lines would be useful for functional spheroid assays. Those which did not form spheroids in STD media or CSC media were eliminated from further study. First, cells from 19 EOC cell lines of various histotypes, including an immortalized endometriosis cell line (12Z), were seeded into 96-well flat bottom ULA plates at multiple cell densities lower than what is standard for spheroid culturing. This is to reduce the amount of autonomous aggregation that spontaneously occurs at high cell densities, and to reduce spheroid size when aggregation does occur. Cells were cultured for 10 days in CSC media, and wells were imaged every 12 h using the IncuCyte S3 live cell imager (Supplementary Table S4).

Based on visual assessment, all cell lines that proliferate in STD media (ES2, KOC-7c, OVTOKO, TOV-21G, RMG-I, and 12Z) also proliferate in CSC media. Unexpectedly, eight OCCC cell lines that do not proliferate in STD media (105C, JHOC-5, SMOV-2, TU-OC-1, OV207, OVISE, RMG-V, EFO21, and EFO27) demonstrated signs of proliferation in CSC media.

**Figure 2:**
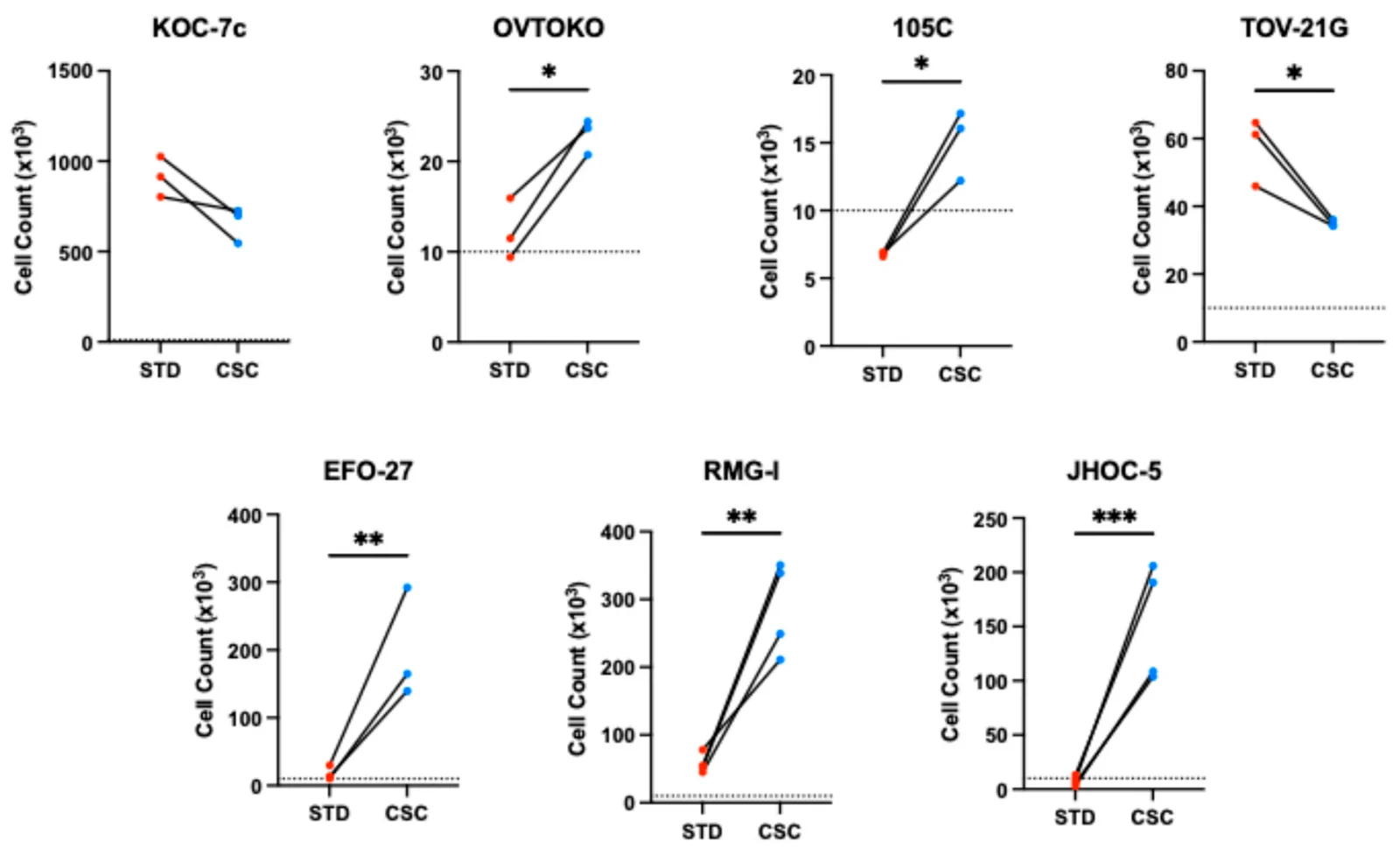
Trypan blue spheroid counting comparison of growth in STD and CSC media. Cells were seeded at a density of 1.0 × 10^4^ cells/well in a 24-well ULA plate for both STD (red) and CSC (blue) media. Spheres from 3 wells were harvested together on day 7 and dissociated with TrypLE for 30 min at 37 °C. TrypLE was neutralized with 50% FBS in PBS, and an equal volume of trypan blue was added prior to counting. The final cell count was divided by 3 to account for the combination of wells during harvesting. The dotted horizontal line represents the starting cell number, 1.0 × 10^4^ cells. Data represent raw cell counts from 3 independent biological replicates; paired *t*-test (* *p* < 0.05, ** *p* < 0.01, *** *p* < 0.001).

To quantitatively assess proliferative capacity in CSC culture conditions and standard (STD) culture conditions, cells from seven OCCC lines were seeded into 24-well ULA plates at low densities and allowed to grow in suspension culture until they were counted on day 7 (Figure 2). These lines were selected to capture a range of *ALDH1A1* mRNA, protein expression, and ALDH activity, with KOC-7C exhibiting the highest ALDH activity by FLOW and 105C as the lowest. The other cell lines included in this assay showed intermediate levels of ALDH activity as shown in Figure 1.

Cell lines that typically proliferate in STD media (KOC-7c, TOV-21G, RMG-I, and OVTOKO) also showed proliferation in CSC media. The RMG-I and OVTOKO cell lines demonstrated higher cell counts in CSC media relative to STD media, showing enhanced proliferation in CSC media. This is contrasted by the KOC-7c and TOV-21G cell lines, which demonstrated higher cell counts in STD media. All the lines that do not proliferate in STD media (105C, JHOC-5, EFO-27) demonstrated higher cell counts in CSC media relative to STD media over the 7-day period. Taken together, proliferative ability in STD media does not predict a cell line’s ability to proliferate in CSC media.

When comparing spheroid morphology, KOC-7c, OVTOKO, TOV-21G, and RMG-I cells form loose clusters, while 105C, JHOC-5, and EFO-27 cells form denser, distinct aggregates. In CSC media, there were no notable differences or changes in morphology relative to STD media morphology, other than a difference in size for spheroids that grew better in one condition over the other (Supplementary Figure S2). Of the lines that grew better in CSC media, both morphologies are represented; however, only the loose clustering spheroid morphology is represented in the lines that grew better in STD media.

**Figure 3:**
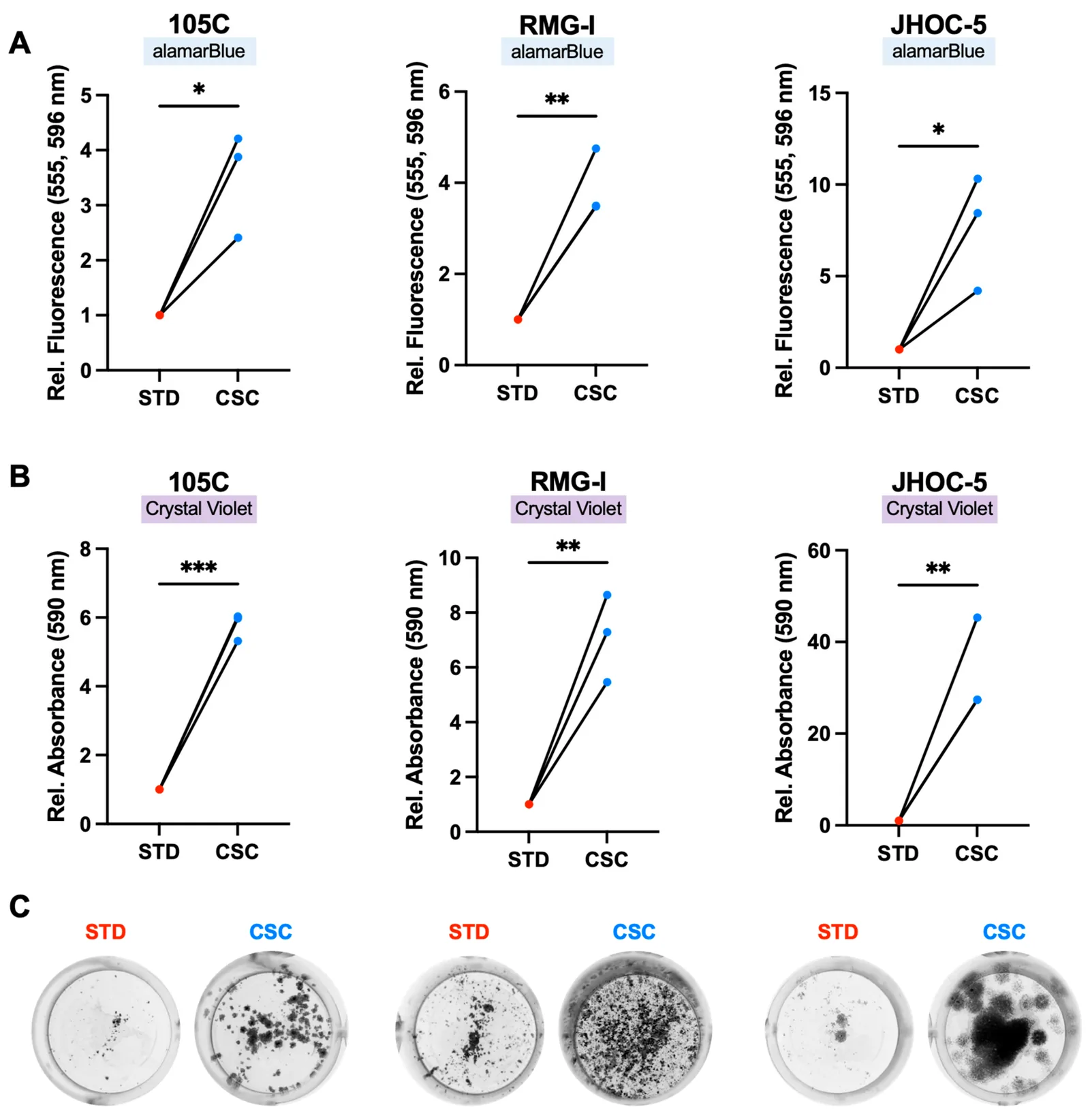
Reattachment of 72 h OCCC cell line spheroids generated in STD and CSC media analyzed using different methods (alamarBlue^TM^, crystal violet, HEMA 3). Cells were seeded at a density of 1.0 × 10^4^ cells/well in a 24-well ULA plate for both STD (red) and CSC (blue) media. After 7 days, spheres were transferred into an adherent 24-well plate and allowed to reattach in spheroid media and FBS-containing media for 72 h. (**A**) Reattached spheroid viability was determined using alamarBlue cell viability reagent, and fluorescence (555, 596 nm) was measured after 4 h. (**B**) Reattached cell biomass was determined using crystal violet staining where absorbance (590 nm) was measured from extracted dye. (**C**) Reattached spheroids stained with Hema 3 showing differences in cell density. Data represent 3 independent biological replicates of STD sphere normalized relative absorbance or fluorescence; paired *t*-test (* *p* < 0.05, ** *p* < 0.01, *** *p* < 0.001).

To confirm our results above and robustly demonstrate enhanced cell viability of CSC media spheres relative to STD media spheres, a spheroid reattachment assay was employed using 105C, RMG-I, and JHOC-5 spheroids. These lines represent low ALDH1A1-expressing lines with strong proliferation in CSC media relative to STD media to generate abundant spheroids for reattachment quantification in this assay. This assay examined a spheroid’s ability to reattach onto adherent tissue culture plates and acts as a surrogate measure of spheroid metastatic potential, as spheroid adhesion is the last step in EOC metastasis. This assay represents a functional assay for spheroid cell viability, as the spheroids that can reattach comprise live cells. After culture in suspension for 7 days in STD or CSC media, spheres were transferred to an adherent culture plate and allowed to reattach for 72 h. The alamarBlue^TM^ assay was then used to quantify reattached sphere cell viability, and crystal violet to quantify reattached cell biomass. Both measures aligned with original cell counts, indicating that these CSC spheres comprise viable cells, a trait commonly attributed to CSCs (Figure 3).

**Figure 4:**
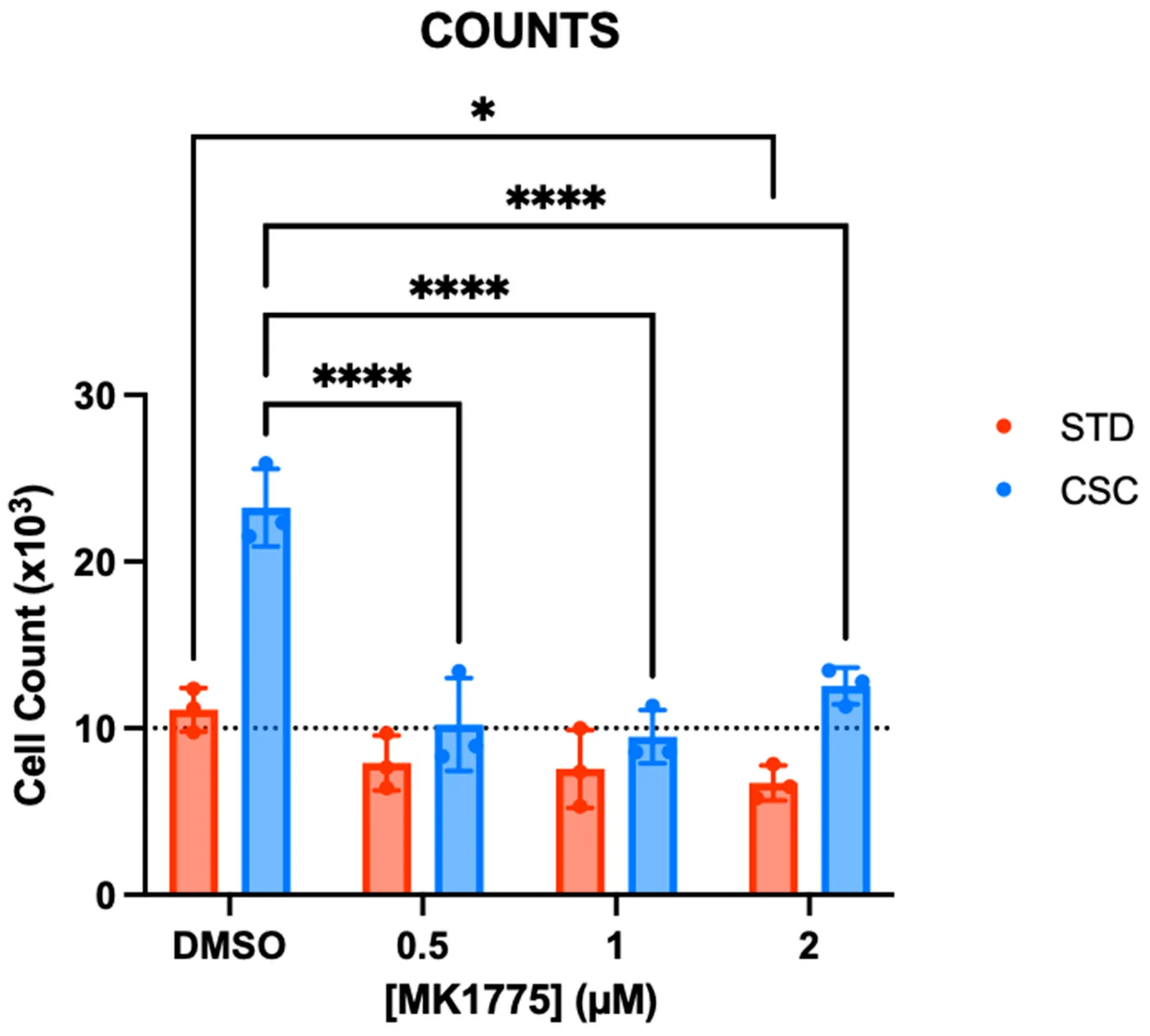
Day 7 105C cell line sphere cell counts in STD and CSC media after 4 days of WEE1 inhibition. Cells were seeded at a density of 1.0 × 10^4^ cells/well in a 24-well ULA plate for both STD (red) and CSC (blue) media. After 3 days, spheres were treated with 0.5 μM, 1, and 2 μM of MK1774 for 4 days, totalling 7 days in culture, after which spheres from 3 wells were harvested together. Spheres were dissociated with TrypLE for 30 min at 37 °C. TrypLE was neutralized with 50% FBS in PBS, and an equal volume of trypan blue was added prior to counting. The final cell count was divided by 3 to account for the combination of wells during harvesting. The dotted line represents 1.0 × 10^4^ cells. Data represent raw cell counts ± SD; two-way ANOVA followed by Tukey’s multiple comparisons test from 3 independent biological replicates (* *p* < 0.05, **** *p* < 0.0001).

Lastly, to further demonstrate OCCC cell line proliferation as spheres in CSC media we chose the 105C cell line because its spheroids are clearly proliferative only in CSC media. We chose biochemical inhibition of WEE1 kinase to demonstrate proliferation of 105C spheroids in CSC media. WEE1 acts as a negative regulator of the cell cycle as it is the primary regulator of the G2/M checkpoint, halting cell cycle progression via the phosphorylation, and subsequent inactivation, of cyclin-dependent kinase 1 at tyrosine 15. When WEE1 activity is lost or impaired, cells would be permitted to enter mitosis prematurely with DNA damage, leading to a loss in genome integrity and ultimately cell death [58,59]. Given this function, highly proliferative cells, which rely heavily on maintaining genome integrity, may be especially sensitive to WEE1 inhibition [58,59]. We hypothesized that proliferating 105C spheroids in CSC media would be preferentially killed by WEE1 inhibition relative to spheroids cultured in STD media. Cells from the 105C line were treated with various concentrations of MK1775 for 4 days after being allowed to grow in suspension for 3 days, for a total time in suspension culture of 7 days. In CSC media, all MK1775 doses significantly decreased spheroid cell number relative to DMSO, whereas spheroids in STD media showed a reduction only at the highest dose, which was closest to its IC50 of 2.6 µM [46], furthering the evidence that 105C spheroids are more viable and proliferate in CSC media relative to STD media (Figure 4).

### 3.3. ALDH1A1 Expression Is Not Aligned with CSC Spheroid Viability and Proliferation

Next, we assessed a subset of CSC markers (ALDH1A1, NANOG, and SOX2) at the protein level to determine if changes in CSC marker expression coincide with changes in proliferation of adherent monolayer cells relative to day 3 and 7 STD and CSC spheroids. Our mRNA analysis demonstrated the highest correlation in NANOG and SOX2 mRNA expression (Figure 1C), influencing our selection of these markers for analysis at the protein level in CSC media. Additionally, these markers are well-established in the literature as relevant to stemness, and we hypothesized they would be selectively expressed in spheroids maintained in CSC media [37,39]. CSC marker expression was assessed in the seven OCCC cell lines used to assess spheroid proliferation in CSC media versus STD media (Figure 2). These lines were also chosen because they generated the necessary mass of spheroids to reproducibly extract adequate protein lysate for Western blot analyses. Hypothetically, cell lines that proliferate better in CSC media would be expected to show increased expression of stemness markers, as increased proliferation would be indicative of selection for enhanced stem-like characteristics from bulk cells.

**Figure 5:**
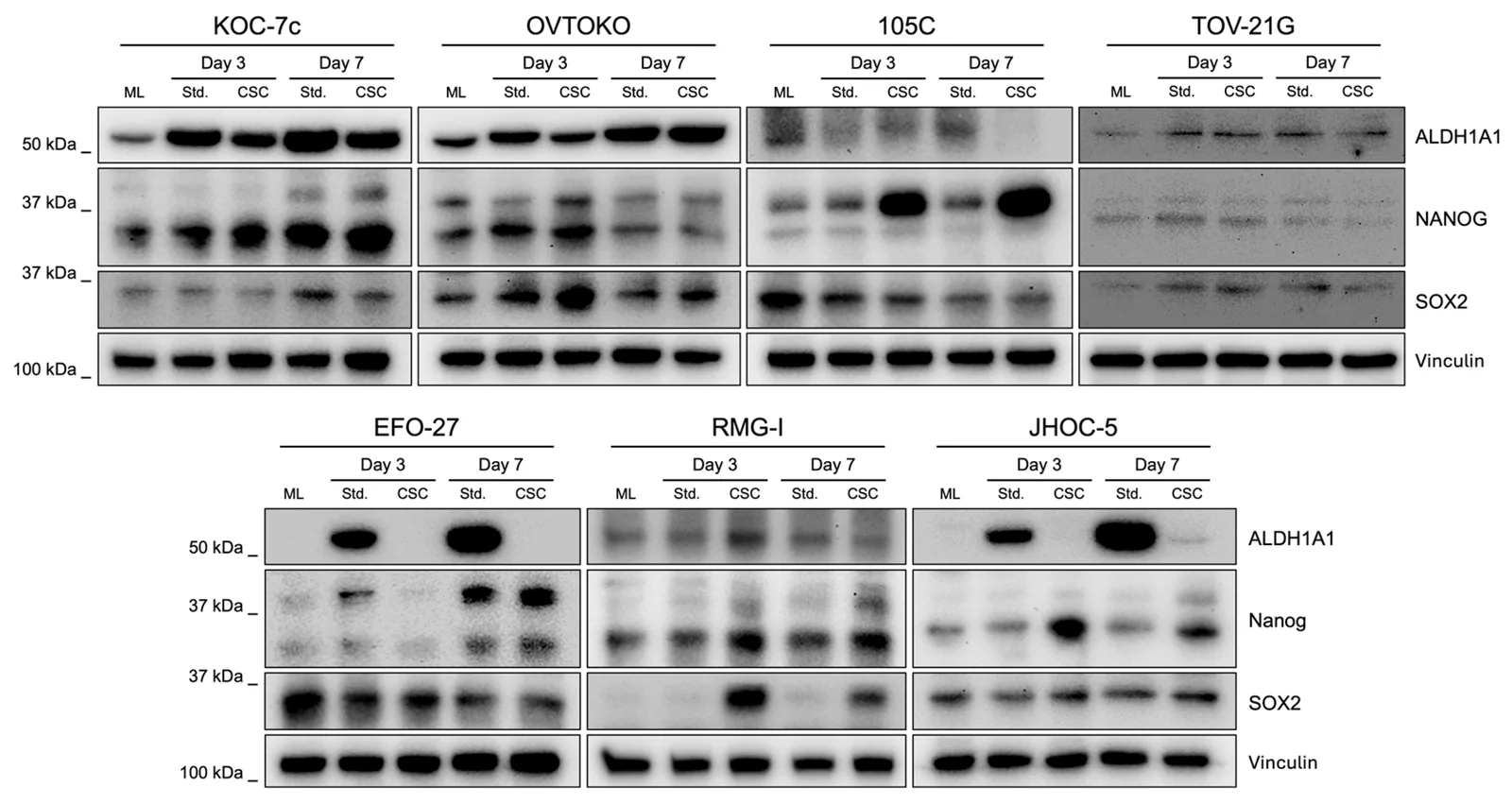
Changes in protein expression of CSC markers in adherent cells, and day 3 and day 7 spheroids cultured in STD and CSC media. Adherent cells were seeded at 0.5–1 × 10^6^ cells per 10 cm plate in STD media. Spheroid (SPH) cells were seeded in 6-well ULA plates at a cell density of 1–2 × 10^5^ cells/well in STD or CSC media. Whole cell protein lysates were collected on day 3 for adherent cells and spheroids, and again on day 7 for spheroids.

Interestingly, while ALDH1A1 is considered a bona fide CSC marker in other cancers, here, its expression is decreased, if not entirely lost, in CSC media in the majority of OCCC lines assessed by Western blotting (Figure 5; Supplementary Figure S3).

Despite all lines demonstrating proliferation in CSC media, only the 105C, RMG-I, and JHOC-5 cell lines demonstrated increased NANOG and SOX2 protein expression that was specific to CSC spheres relative to STD spheres. KOC-7c cells appear to demonstrate a time-dependent increase in marker expression, regardless of media, while TOV-21G cells experience a modest increase in expression of these marks in spheroid culture relative to adherent culture. Despite demonstrating proliferative ability in CSC media, there was no CSC sphere-specific increase in marker expression in the OVTOKO and EFO-27 cell lines. OVTOKO spheroids demonstrated NANOG and SOX2 expression in day 3 spheroids in both media, and EFO-27 spheres exhibited higher NANOG expression in day 7 spheroids in both media.

These observations do not necessarily preclude the possibility of stem-like cell enrichment, but if there is enrichment, those cells were not delineated by expression of the assayed CSC marks. This result emphasizes the need for a more global analysis to identify the basis of CSC spheroid growth advantage under CSC-promoting conditions.

### 3.4. Drug-Resistant Cells Appear to Be Enriched for CSCs

CSCs are inherently resistant to chemotherapy treatment through a variety of mechanisms, including increased presence of efflux pumps, which expel exogenous drugs, as well as high-efficiency DNA repair mechanisms in response to DNA damage [60,61,62,63,64,65,66]. Tumour CSCs are also slowly proliferative and are often in a state of quiescence, which further limits the efficacy of chemotherapeutics as their primary method of action relies on active cell division [19,67,68]. Therefore, we hypothesized that chemotherapy-resistant OCCC cell lines will exhibit enhanced CSC characteristics relative to their chemotherapy-sensitive parental counterparts and provide unique reagents for OCCC CSC enrichment and analyses. Additionally, the acquisition of drug resistance mirrors the current clinical challenges with chemo-resistant tumours, a consequence commonly attributed to CSCs [69,70].

To determine if chemotherapy treatment can enrich or select for CSCs from a bulk cell population, three OCCC cell lines (OVTOKO, TOV-21G, and 105C) were selected for analysis. The OVTOKO cell line was chosen due to its strong ALDH1A1 mRNA and protein expression, and high proportion of cells with high ALDH family activity; the TOV-21G and 105C cell lines were selected due to low ALDH1A1 expression and ALDH activity. We hypothesized that those lines with low ALDH1A1 levels (105C and TOV-21G) would show significant enhancement of CSC properties in CSC media when made drug-resistant, as suggested by the literature. Bulk cell sensitivity was assessed using paclitaxel, a standard-of-care chemotherapeutic for EOC treatment with low efficacy in OCCC, and AZD-8055, a selective mTOR inhibitor that was selected due to the frequency of mutations in the PI3K signalling pathway in OCCC tumours [4]. These drugs contrast in their mechanism of action, with paclitaxel promoting a cytotoxic response and AZD-8055 promoting a cytostatic response [71,72]. Of the three cell lines examined, the OVTOKO cell line is the only line that was not chemo-naïve, as the patient underwent three rounds of cyclophosphamide, adriamycin, and platinum (CAP) combination chemotherapy prior to cell collection [73]. The TOV-21G cell line is both chemotherapy and radiation naïve, while the 105C cell line is not radiation naïve, as it was collected at the first instance of disease relapse after abdominal radiation treatment [74,75]. Drug-resistant OCCC cell lines for these drugs have not been previously generated, with the exception of a paclitaxel-resistant TOV-21G [76].

**Figure 6:**
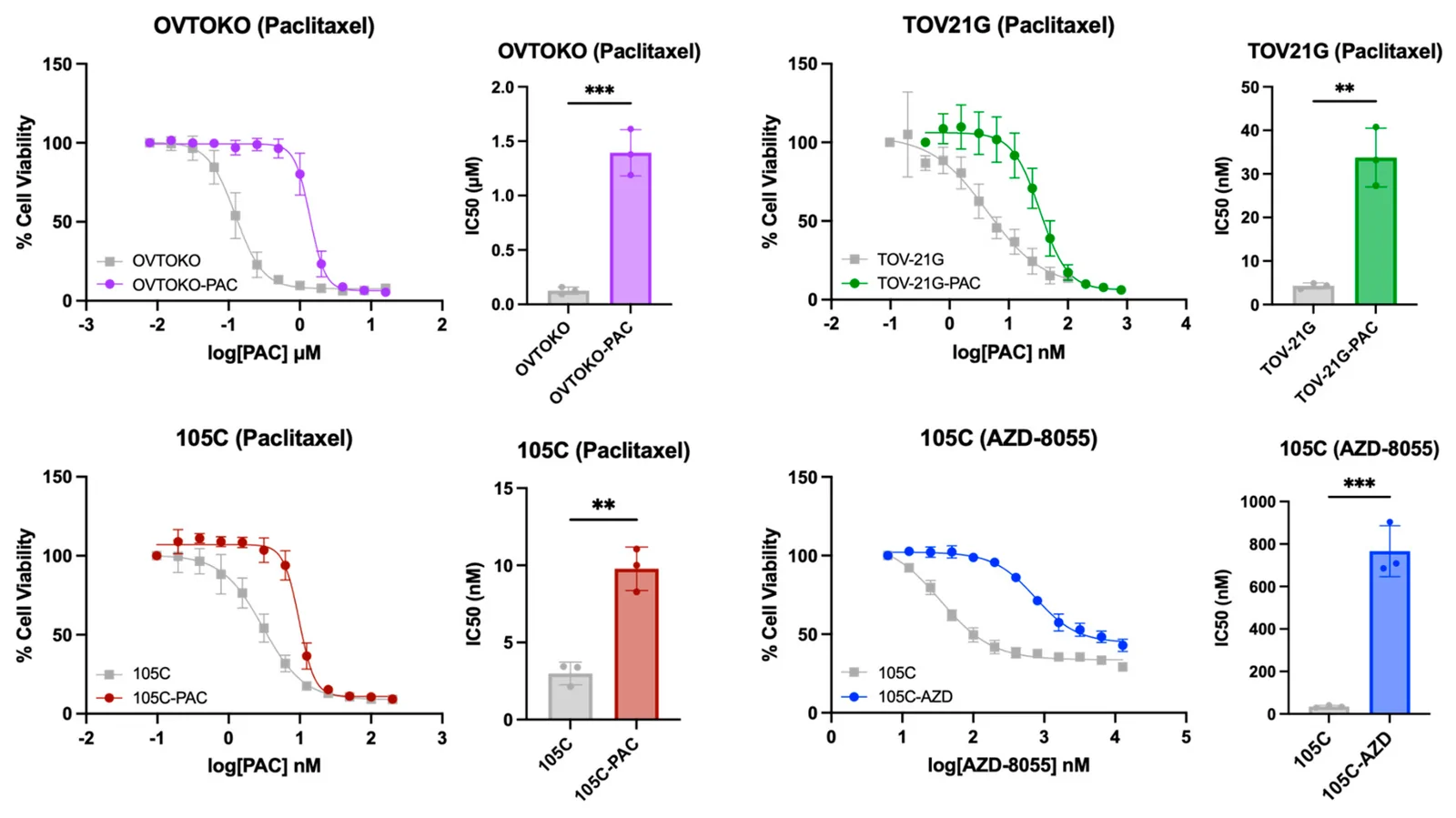
Cell viability of parental and drug-resistant OVTOKO, TOV-21G and 105C cell lines treated with paclitaxel or AZD-8055. IC50 curves for OVTOKO, TOV-21G, and 105C parental cell lines (grey) and drug-resistant (colour) cell lines in adherent culture conditions in STD media. Cells were seeded in adherent 96-well plates at a density of 3000 cells/well (OVTOKO and OVTOKO-PAC), 1500 cells/well (TOV-21G and TOV-21G-PAC), and 2000 cells/well (105C, 105C-PAC, and 105C-AZD) in triplicate wells and treated 24 h after seeding for 72 h. Cell viability was determined using alamarBlue cell viability reagent, and fluorescence (555, 596 nm) was measured after 4 h. IC50 values were calculated using Prism GraphPad Prism. Data represent 3 independent biological replicates; bar graphs represent the mean difference in IC50 ± SD; unpaired t test (** *p* < 0.01, *** *p* < 0.001).

OCCC cell lines were made resistant to paclitaxel or AZD-8055 to determine if chronic drug exposure results in CSC-enriched cell populations in resistant cell lines relative to parental cell lines. The drug-resistant lines were expected to provide a highly enriched source of CSCs for further investigation relative to the parental OCCC lines. Over a period of 5–8 months, cells were chronically exposed to increasing drug concentrations, after which changes in IC50 values were assessed (Figure 6; Table 4).

**Figure 7:**
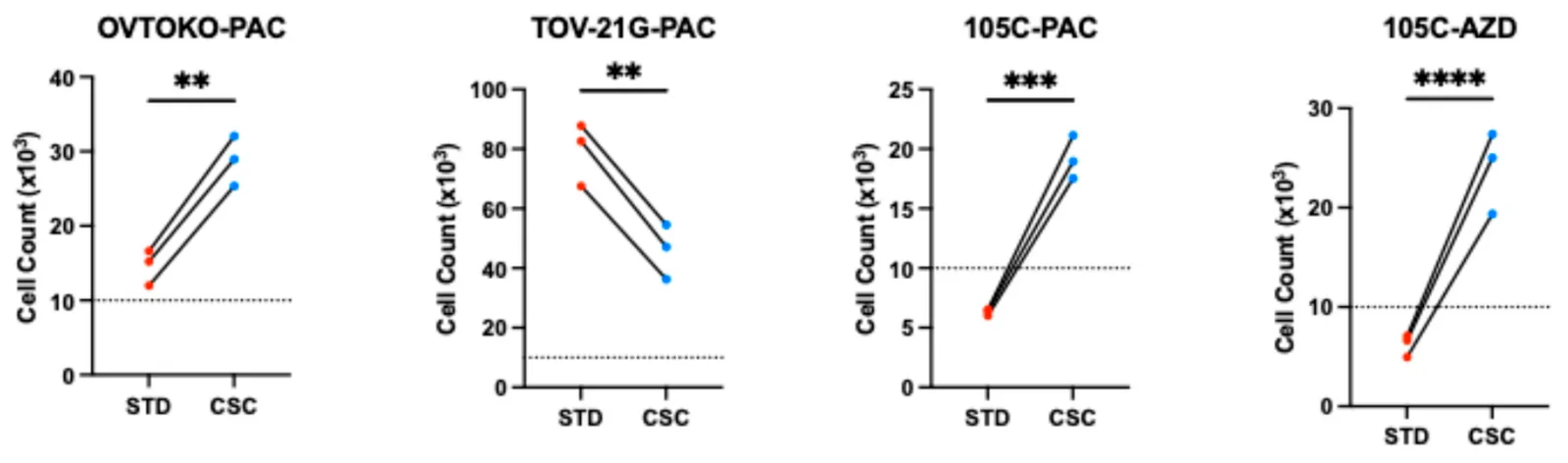
Trypan blue spheroid counting comparison of resistant cell lines’ growth in STD and CSC media. Cells were seeded at a density of 1.0 × 10^4^ cells/well in a 24-well ULA plate for both STD (red) and CSC (blue) media. Spheres from 3 wells were harvested together on day 7 and dissociated with TrypLE for 30 min at 37 °C. TrypLE was neutralized with 50% FBS in PBS, and an equal volume of trypan blue was added prior to counting. The final cell count was divided by 3 to account for the combination of wells during harvesting. The dotted line represents the starting cell number, 1.0 × 10^4^ cells. Data represent raw cell counts from 3 independent biological replicates; paired *t*-test (** *p* < 0.01, *** *p* < 0.001, **** *p* < 0.0001).

To determine if the acquisition of drug resistance led to enhanced proliferation of spheres in CSC media, we assessed proliferative capacity and viability in STD and CSC media. Cells from the resistant and parental lines were seeded at low densities and allowed to grow in suspension culture until they were assessed on the seventh day by trypan blue exclusion cell counting. Cell count trends in resistant lines remained similar to the parental cell lines (Figure 7). Parental lines that proliferate better in CSC media relative to STD media (105C and OVTOKO) produced resistant lines (105C-PAC, 105C-AZD, OVTOKO-PAC) that also proliferate better in CSC media.

Since parental 105C and OVTOKO cell lines responded to the stem-promoting cues in CSC media with elevated proliferation, it follows that their resistant counterparts maintained that advantage. The reverse was also true as both parental and resistant TOV-21G cell lines proliferated better in STD media relative to CSC media. This result could be interpreted that TOV-21G cells are less capable of exploiting stemness-promoting cues in the CSC media or may express factors that are unfavourable to spheroid growth under CSC conditions. As parental TOV-21G cells were not better adapted to CSC media, their resistant counterpart (TOV-21G-PAC) did not gain that advantage.

**Figure 8:**
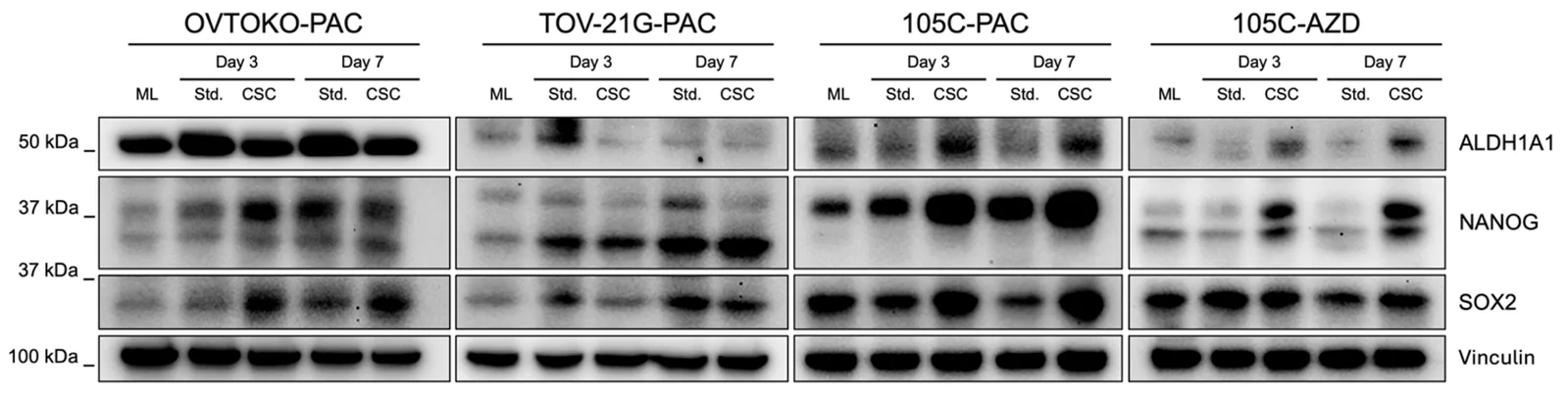
Changes in protein expression of CSC markers in adherent cells, day 3 and day 7 drug-resistant cell line spheroids cultured in STD and CSC media. Cells were seeded in 6-well ULA plates at a cell density of 1–2 × 10^5^ cells/well in STD or CSC media. Adherent cells were seeded at 0.5–1 × 10^6^ cells per 10 cm dish in STD media. Whole cell protein lysates were collected on day 3 for adherent cells and spheroids, and again on day 7 for spheroids.

Next, protein levels of ALDH1A1, NANOG, and SOX2 were assessed in bulk adherent cells and in day 3 and 7 STD and CSC spheres of drug-resistant cell lines. This was to determine if the acquisition of drug resistance influenced CSC marker expression profile in resistant cell lines relative to parental cell lines (Figure 8; Supplementary Figure S4).

The parental OVTOKO cell line demonstrated a reduction in ALDH1A1 expression in CSC media relative to STD media, as did OVTOKO-PAC (Figure 5). However, while parental OVTOKO cells demonstrated increased SOX2 expression that was not isolated or specific to either media condition, OVTOKO-PAC cells showed a CSC-specific increase in SOX2. Parental TOV-21G cells showed negligible levels of the assessed CSC marks; however, TOV-21G-PAC cells showed a time-dependent increase in NANOG and SOX2, and an increase in ALDH1A1 in day 3 STD media spheroids. Parental 105C cells consistently show elevated NANOG expression in CSC media, as did 105C-PAC and 105C-AZD. Additionally, 105C-PAC and 105C-AZD lines also demonstrated CSC-specific increases in ALDH1A1 and SOX2. While this is the only instance of ALDH1A1 expression enrichment in CSC media relative to STD media from any cell line examined in this study, this apparent enrichment was not consistent (Supplementary Figure S4).

### 3.5. OCCC CSC Spheroids Display Molecular Characteristics of Normal SCs and CSCs

**Figure 9:**
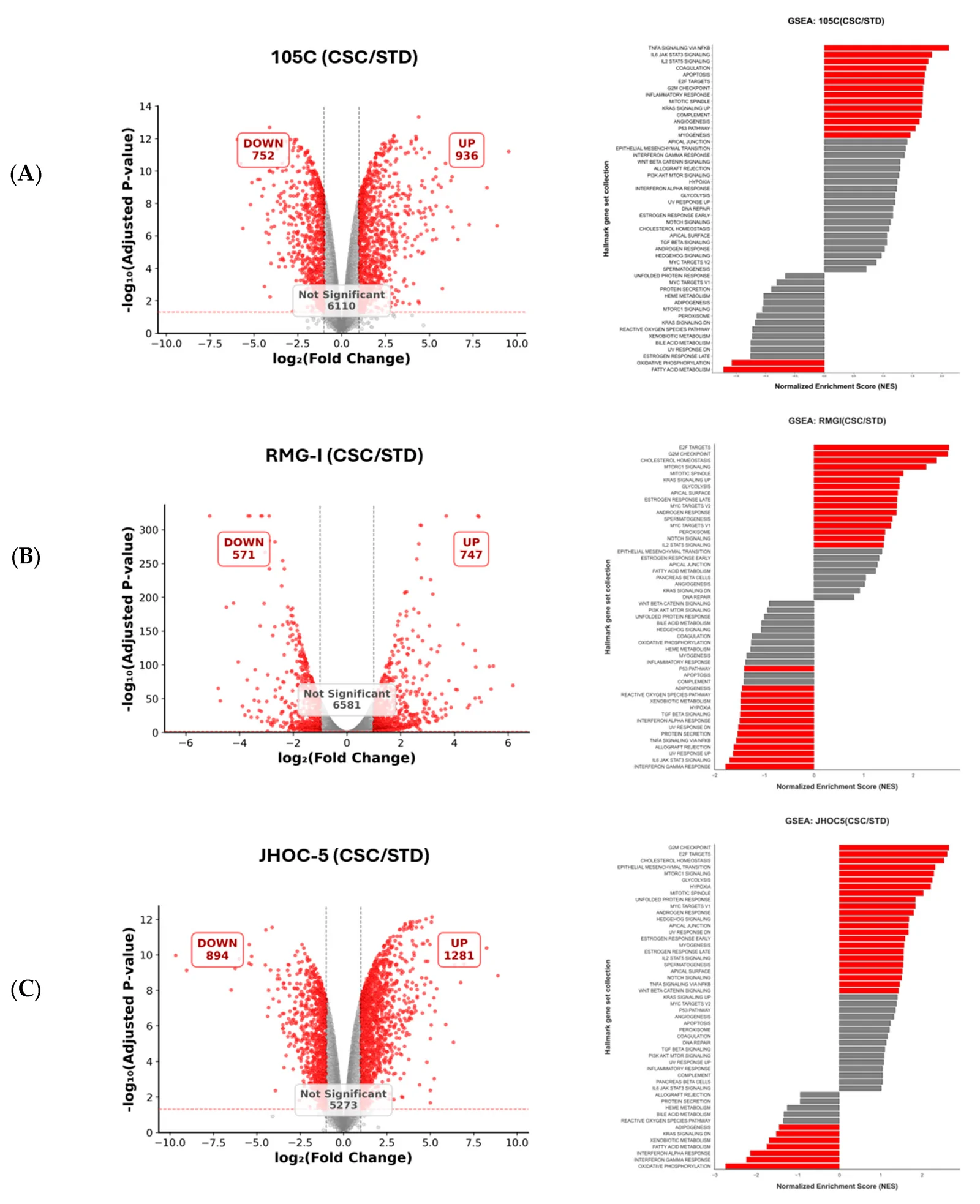
Per-line transcriptional response to cancer-stem-cell versus standard media. (**A**) Principal component analysis of log2 CPM-normalized RNA-seq counts across all 16 samples (*n* = 2 per cell line × media combination). PC1 vs. PC2 (**A**) captures 40.0% and 27.3% of total variance respectively; PC2 vs. PC3 resolves an additional 10.1% of variance on PC3-showing additional variance associated with media type. Marker shape encodes culture media; marker colour encodes cell line and drug treatment. (**B**,**C**) Each row presents one cell line (top to bottom: 105C, JHOC-5, RMG-I), with two complementary views of its CSC-vs-STD response. (**B**) Volcano plots of all genes tested by limma-voom for the per-line CSC-vs-STD comparison. Each point is one gene; significantly differentially expressed genes (adj. *p* < 0.05) above |log2FC| > 1 are shown in red, non-significant genes (or genes where |log2FC| < 1) are shown in grey. Per-line totals are annotated on the plot: DOWN = significant with logFC < 0, **UP** = significant with logFC > 0, Not Significant = adj. *p* ≥ 0.05. (**C**) GSEA Hallmark pathway enrichment for the matched comparison. Pre-ranked GSEA was performed via the Broad Institute’s GSEA using Hallmark gene-set. Each horizontal bar represents one Hallmark gene set, with bar length proportional to the normalized enrichment score (NES). Red bars mark FDR-significant sets in the positive (pathway up in CSC media) and negative (pathway down in CSC media) directions; grey bars indicate non-significant enrichment. Sets are sorted by NES.

To define the transcriptional changes associated with the differential spheroid growth observed in CSC media, we performed bulk RNA-seq on day-7 OCCC spheroids cultured in CSC or STD media (*n* = 2 biological replicates per cell line and medium). We chose the 105C, JHOC-5 and RMG-I cell lines for these analyses because they represent low ALDH1A1-expressing lines yet displayed strong spheroid proliferation in CSC media relative to STD media. Moreover, we observed CSC media-specific expression of stem-associated markers or reduced expression of *ALDH1A1* in these lines, meaning that they exhibited distinct responses to CSC media in spheroid form. Specifically, 105C up-regulated *NANOG*, JHOC-5 up-regulated *NANOG* and showed loss of *ALDH1A1*, and RMG-I up-regulated *SOX2* (Figure 5). Therefore, we hypothesized that large differences in gene expression would be uncovered by comparing the STD versus CSC spheroid transcriptomes. We also expected to discover commonalities in gene expression due to CSC media enrichment of the CSC phenotype across cell line spheroids. Principal component analysis of all sequenced samples showed that cell line identity was the dominant source of transcriptional variance, separating the lines along the first and second principal components (40.0% and 27.3% of variance), whereas the CSC-versus-STD media contrast resolved on the third principal component (10.1% of variance) (Figure 9A). Although each cell line retains a distinct baseline transcriptome, CSC media imposes a reproducible, media-specific transcriptional shift within each line.

We next examined the per-line response by differential expression analysis for the three lines (105C, JHOC-5, and RMG-I) in both media. We found that CSC media drove extensive, bidirectional transcriptional remodelling in every line, with large numbers of genes significantly induced and repressed and a broadly symmetric split between up- and down-regulation (Figure 9B). The scale of this response indicates that CSC media reprogrammes the spheroid transcriptome globally, rather than perturbing a small set of genes. To identify the biological programmes driving this response, we performed gene set enrichment analysis (GSEA) against the MSigDB Hallmark collection for each line (Figure 9C). Gene sets associated with proliferation, comprising E2F targets, the G2/M checkpoint, and mitotic spindle, were concordantly enriched in CSC spheroids across all three lines, with MYC targets and mTORC1 signalling additionally enriched in JHOC-5 and RMG-I cell lines. Both of these lines also showed enriched expression of genes important for glycolysis and cholesterol homeostasis (and JHOC-5 alone, hypoxia and epithelial-to-mesenchymal transition), whereas oxidative phosphorylation was significantly de-enriched in 105C and JHOC-5 cell lines. Strikingly, inflammatory and cytokine-signalling programmes diverged by line: TNFα signalling via NF-κB and IL6-JAK-STAT3 were among the most strongly enriched sets in the 105C cell line but were de-enriched in RMG-I cells. CSC media therefore elicits a shared cell proliferation programme overlaid with line-specific metabolic and inflammatory responses.

**Figure 10:**
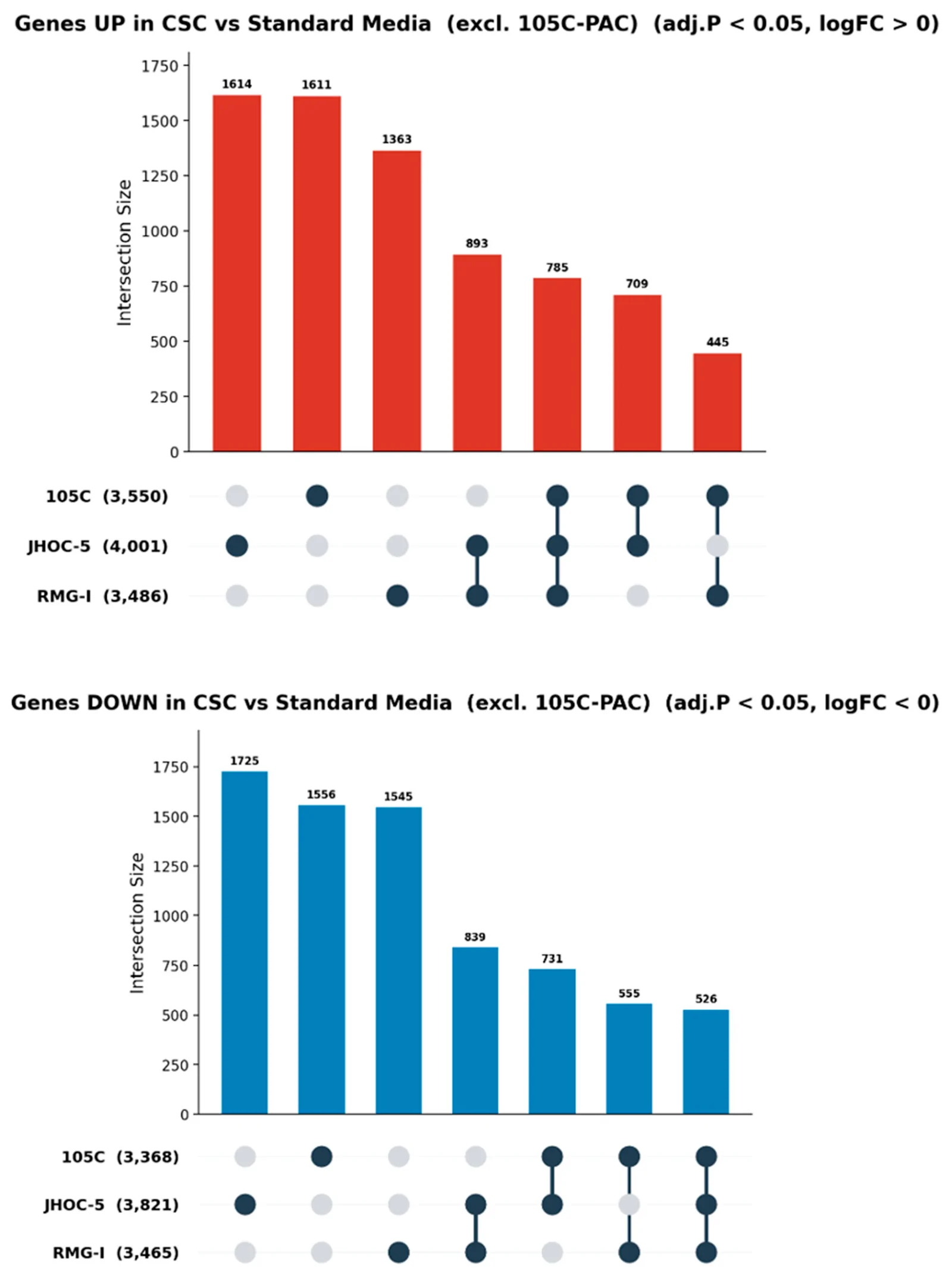
A shared three-line cancer-stem-cell-media signature defined by intersection of per-line differentially expressed genes. Three-way UpSet plots of significantly differentially expressed genes (limma-voom; adjusted *p* < 0.05, Benjamini-Hochberg) UP in CSC media (logFC > 0; red bars) and DOWN in CSC media (logFC < 0; blue bars). The 105C paclitaxel-resistant line is excluded from this consensus analysis to isolate media-driven differential expression. Each bar represents the number of genes unique to the set of cell lines such that they do not overlap with any of the other gene sets within the bar graphs. The dot matrix below each bar identifies the cell-line membership of that intersection (filled = member of the indicated set, open = non-member); connecting lines join filled dots within multiset intersections. The number in parentheses next to each row label is that cell line’s count of significantly DE genes in the panel shown (UP-significant genes in the UP panel, DOWN-significant genes in the DOWN panel). The all-three-lines intersection (filled dots in all three rows) defines the 3-line core consensus signature: 785 genes consistently UP and 526 genes consistently DOWN in CSC media across all three lines.

To uncover the gene expression changes induced by CSC media relative to STD media and shared across the three OCCC cell lines, we intersected the per-line differentially expressed genes (adj. *p* < 0.05; excluding the paclitaxel-resistant condition) so that the signature reflects media-induced changes rather than drug effects. This defined a three-cell line core of 785 genes concordantly up-regulated and 526 genes concordantly down-regulated in CSC media across the 105C, JHOC-5, and RMG-I cell lines (Figure 10). Because concordant differential expression across three independent cell lines is far less likely to reflect line-specific artefacts than any single-line result, this 1,311-gene consensus signature provides the most robust molecular description of the CSC-media response in this dataset as applied to OCCC cell lines in spheroid form (Supplementary Table S5).

To test whether this consensus signature corresponds to a recognized stem-cell programme, we assessed its overlap with 20 curated stemness gene sets by one-sided Fisher’s exact test, using the genes testable in all three lines as the background. The 785-gene up-signature was significantly enriched in 10 of the 20 sets, whereas the 526-gene down-signature was enriched in none (Supplementary Tables S6 and S7). The enriched sets included embryonic stem-cell signatures (BENPORATH ES, WONG ESC core, Bhattacharya ESC), transcriptional targets of NANOG and SOX2, embryonic stem-cell transcription-factor and iPSC signatures, and adult/mesenchymal stem-cell signatures, consistent with the induction of a stem-like transcriptional state rather than a generic stress response.

Finally, to identify the cell type most resembling the shared up-regulated genes, we performed over-representation analysis against the PanglaoDB cell-type signature collection, which returned pluripotent stem cells as the top-ranked cell type (Supplementary Table S8). Taken together, these analyses show that OCCC cells maintained as spheroids in CSC media adopt a transcriptional state enriched for proliferative and embryonic stem-cell programmes, most resembling pluripotent stem cells, indicating that CSC-media spheroids are enriched for cells bearing the molecular characteristics of OCCC cancer stem cells.

### 3.6. ALDH1A1 Is Not a Marker of OCCC Spheroid Cells Cultured CSCin CSC Media

**Figure 11:**
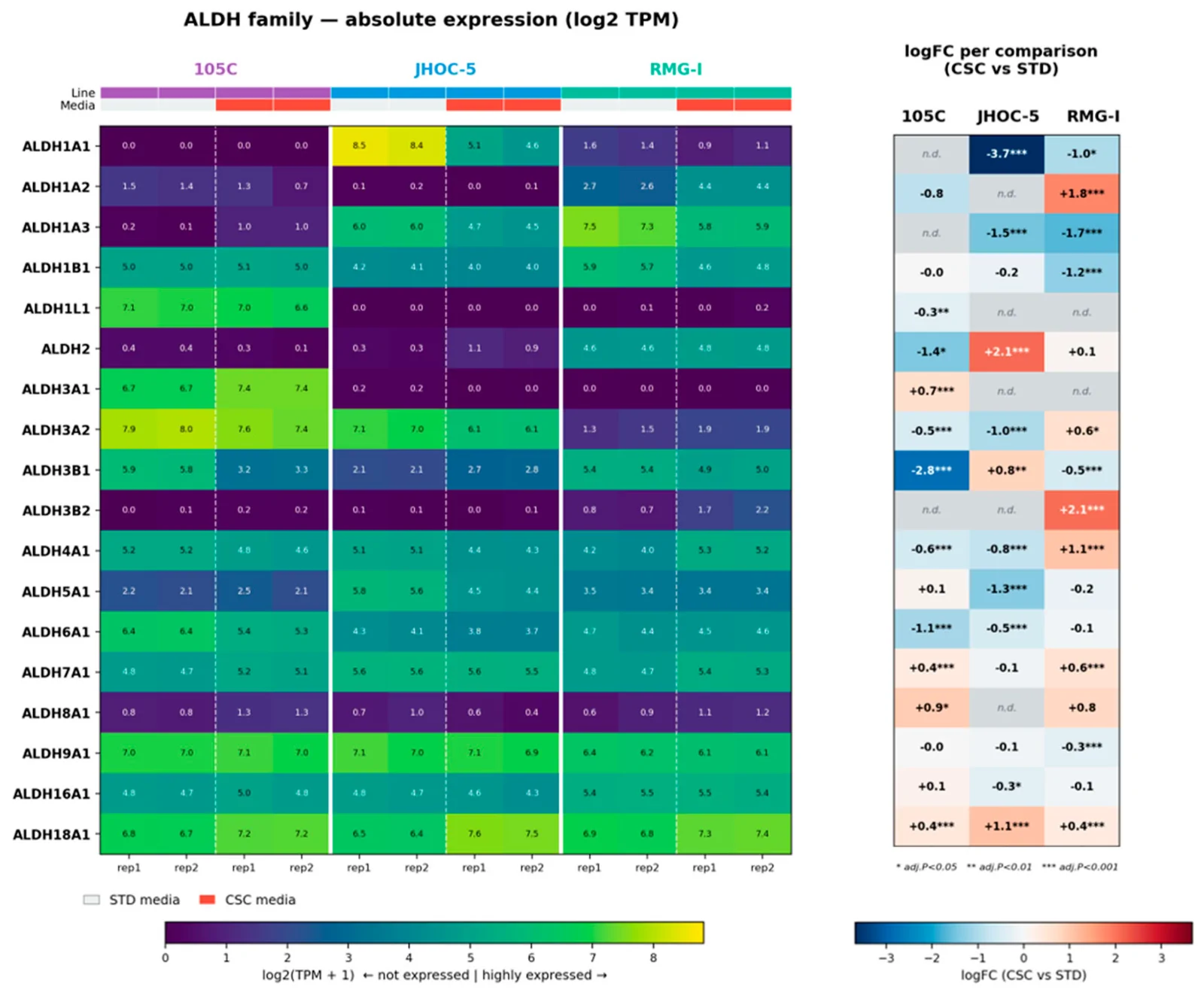
ALDH gene family expression in 105C, JHOC-5, and RMG-I spheroids maintained in CSC versus STD media. (**Left panel**) Absolute expression of 18 of the 19 protein-coding ALDH family members, displayed as log2(TPM + 1) across all 12 RNA-seq samples (*n* = 2 per cell-line × media combination). TPM values were derived from the limma-voom-normalized log2 CPM matrix using Ensembl canonical transcript lengths. Annotation bars at top encode cell-line identity (purple = 105C, blue = JHOC-5, teal = RMG-I) and culture media (light grey = standard/STD, red = cancer stem cell/CSC). (**Right panel**) Per-cell-line CSC-vs-STD log2 fold change for each ALDH gene from the corresponding limma-voom DE comparison. Significance asterisks: * adj. *p* < 0.05, ** adj. *p* < 0.01, *** adj. *p* < 0.001 (Benjamini–Hochberg-corrected within each per-line DE comparison).

Having established that CSC-media spheroids adopt a stem-cell-like transcriptional state, we asked whether the canonical CSC marker ALDH1A1, and the wider ALDH family, was transcriptionally induced under these conditions. We profiled the ALDH gene family across the three cell lines in spheroids generated in STD and CSC media, comparing absolute expression with the per-line CSC-versus-STD response (Figure 11); 18 of the 19 human ALDH family members were detectably expressed and are shown (ALDH1L2 fell below the low-expression detection threshold in all samples and was excluded from downstream analysis).

The ALDH family showed no coordinated induction of a detoxification- or stemness-associated member in OCCC cell-line-derived CSC-enriched spheroids. The only ALDH gene significantly and concordantly up-regulated across all three lines was ALDH18A1 (log2FC +0.4 to +1.1; adj. *p* < 0.001 in each line), which encodes a Δ1-pyrroline-5-carboxylate synthase of proline biosynthesis rather than an ALDH-activity stemness marker (Figure 11). The canonical marker *ALDH1A1* was not induced in any line. In JHOC-5 cells, where *ALDH1A1* is robustly expressed is STD spheroids, it was significantly repressed in CSC spheroids (log2FC = −3.7), paralleling the loss of ALDH1A1 protein observed in JHOC-5 CSC spheroids (Figure 5); *ALDH1A3*, the other ALDH most frequently cited as an ovarian cancer-CSC marker, was likewise repressed where it is expressed (JHOC-5 and RMG-I; log2FC −1.5 and −1.7). In the 105C line, *ALDH1A1* fell below the expression-detection threshold, and in RMG-I cells it was expressed only just above background (≈1 log2 TPM), where its nominally significant down-regulation (log2FC = −1.0) carries little biological weight. The absence of any coordinated induction of detoxification- or stemness-associated ALDH members, together with the repression of *ALDH1A1* and *ALDH1A3* where they are expressed, indicates that *ALDH1A1* is unlikely to serve as a transcriptional marker of cancer stem cells in CSCs of established human OCCC cell lines.

### 3.7. RNA-Seq Analysis of Parental vs. Drug-Resistant 105C Cell Lines

**Figure 12:**
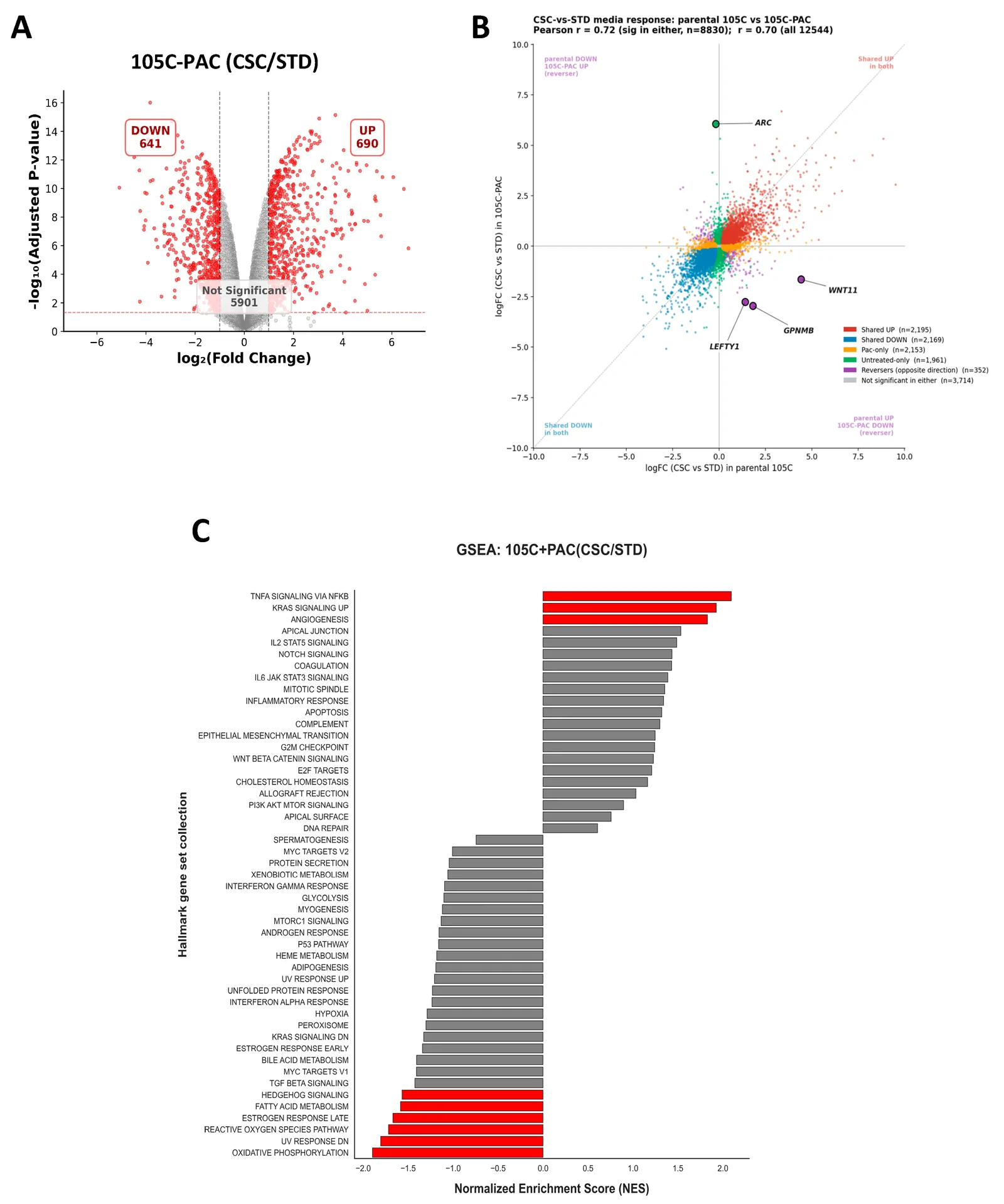
Transcriptional response to CSC media spheroids in the paclitaxel-resistant 105C-PAC line and its concordance with parental 105C spheroids. 105C-PAC is a stably paclitaxel-resistant subline of 105C; no drug was applied to the sequenced cells, so all comparisons reflect cell-line and media differences. (**A**) Volcano plot of the 105C-PAC CSC-vs-STD limma-voom comparison. X-axis: log2 fold change (positive = up in CSC media); Y-axis:-log10 of the Benjamini–Hochberg-adjusted *p*-value. Genes significant at adj. *p* < 0.05 and |log2 fold change| > 1 are shown in red and counted as UP (690) or DOWN (641); all other genes are grey. (**B**) Gene-by-gene comparison of the CSC-vs-STD response between the parental and resistant lines. Each point is one of 12,544 genes tested in both lines; axes give the CSC-vs-STD log2 fold change in parental 105C (x) and in 105C-PAC (y), and the dashed diagonal marks an identical response. Genes are coloured by significance category (limma-voom adj. *p* < 0.05, applied independently to each line): Shared UP (red, *n* = 2195) and Shared DOWN (blue, *n* = 2169), significantly changed in the same direction in both lines; 105C-PAC-specific (green, *n* = 2153) and parental-specific (orange, *n* = 1961), significant in only one line; reversers (purple, *n* = 352), significant in both but in opposite directions; and not significant in either (grey, *n* = 3714). The CSC-media responses of the two lines are strongly correlated (Pearson r = 0.70 across all genes; r = 0.72 among the 8830 significant in either line). (**C**) Pre-ranked Hallmark GSEA of the 105C-PAC CSC-vs-STD comparison; bar length is the normalized enrichment score (NES) and red bars are statistically significant. Positive NES marks Hallmark sets enriched among genes up-regulated in CSC media; negative NES, sets enriched among down-regulated genes.

To determine the molecular basis of paclitaxel resistance and whether it alters the CSC media response, we profiled the paclitaxel-resistant subline 105C-PAC in both CSC and standard media (*n* = 2 per medium) alongside its parental 105C line. 105C-PAC is a stably resistant line; no drug was applied to the sequenced cells, so every comparison reflects cell-line and media differences rather than acute drug treatment. CSC media drove a robust transcriptional response in 105C-PAC, with 690 genes significantly induced and 641 repressed (adj. *p* < 0.05 and |log2 fold change| > 1; Figure 12A, Supplementary Table S9).

Next, we chose to represent these data as previously performed [46,77] using the ratio of expression changes: [105C(STD/CSC)]/[105CPAC(STD/CSC)]. This approach allows a pictorial demonstration of the gene expression changes which occurred in the spheroids from each of these lines in CSC media, both in direction and scale, which permits between line comparisons. This analysis allows us to determine which genes show a differential response in spheroid cells cultured in CSC media. This graph (Figure 12B) shows the fold change in gene expression between STD and CSC media for the parental and paclitaxel-resistant 105C spheroids presented as ratios. The shared genes expression changes are shown in the red and blue quadrants (2195 genes up and 2169 genes down), while the purple dots denote the gene expression changes significantly altered in opposite directions when the cell lines are compared (352 genes). Examples of the genes uniquely altered in CSC spheroids in a cell-line-specific manner are indicated by purple dots and include stemness- and WNT-pathway regulators: *WNT11*, *LEFTY1* and *GPNMB* (Glycoprotein non-metastatic melanoma protein B) are induced by CSC media in parental 105C yet repressed in 105C-PAC, while ARC was strongly induced by CSC media only in the resistant line (highlighted, Figure 12B). This pattern indicates that paclitaxel resistance superimposes a distinct, partially discordant transcriptional programme on an otherwise largely conserved core response to the CSC-media environment.

We also assessed the expression of the ALDH family of genes in 105C parental cell line spheroids maintained in STD and CSC media to the paclitaxel-resistant cell line spheroids. The only ALDH family member, in the paclitaxel-resistant line, which exhibited a significant increase in mRNA level in CSC media was *ALDH8A1* (Supplementary Figure S5), which is primarily involved in amino acid breakdown [78]. *ALDH1A1* mRNA levels did not change upon spheroid culture in CSC media. *ALDH3B1* mRNA levels increased significantly in CSC spheroids of both lines. It plays a role in the detoxification of aldehydes by oxidation [79].

Hallmark gene set enrichment analysis (GSEA) of the 105C-PAC response recapitulated the parental programme, enriching inflammatory and angiogenic signatures (TNFα signalling via NF-κB, KRAS signalling, angiogenesis) among CSC-induced genes and oxidative phosphorylation, reactive-oxygen-species, and fatty-acid-metabolism programmes among CSC-repressed genes (Figure 12C). The resistant line therefore mounts essentially the same media-responsive transcriptional programme as its parent.

**Figure 13:**
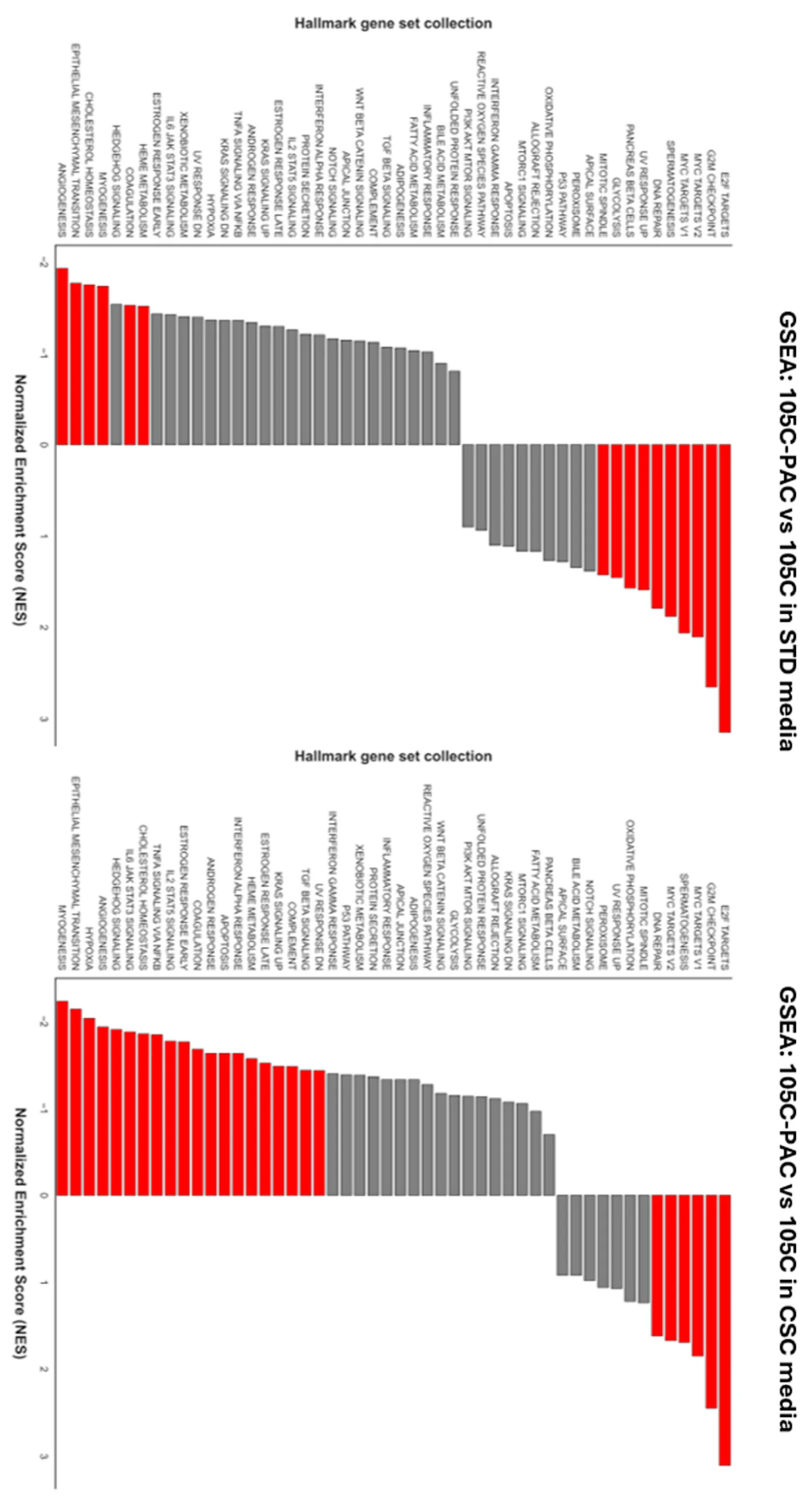
Hallmark pathway differences between paclitaxel-resistant 105C-PAC and parental 105C, by medium. Pre-ranked Hallmark GSEA of the 105C-PAC-versus-parental limma-voom contrast, computed separately within STD media (left) and CSC media (right). Bar length is the NES; positive NES (rightward) marks Hallmark sets enriched among genes expressed more highly in 105C-PAC than parental, negative NES (leftward) marks sets enriched among genes lower in 105C-PAC. Red bars are statistically significant.

We next asked how the resistant line differs from parental at the cell-line level, comparing 105C-PAC with parental 105C within each medium by Hallmark GSEA (Figure 13). The two contrasts were highly consistent. In both STD and CSC media, the resistant line was enriched for proliferation-associated programmes (E2F targets, the G2/M checkpoint, MYC targets, and DNA repair) and de-enriched for epithelial-to-mesenchymal transition, angiogenesis, and cholesterol homeostasis. In CSC media the resistant line was additionally de-enriched, relative to parental, for the inflammatory and hypoxic programmes that CSC media itself induces (TNFα signalling via NF-κB, IL6-JAK-STAT3 signalling, interferon-α response, and hypoxia); this between-line difference is distinct from, and not in conflict with, the induction of those same programmes by CSC media within each line (Figure 12C). The dominant, media-independent feature distinguishing the resistant line is thus a shift toward a proliferative transcriptional state.

**Figure 14:**
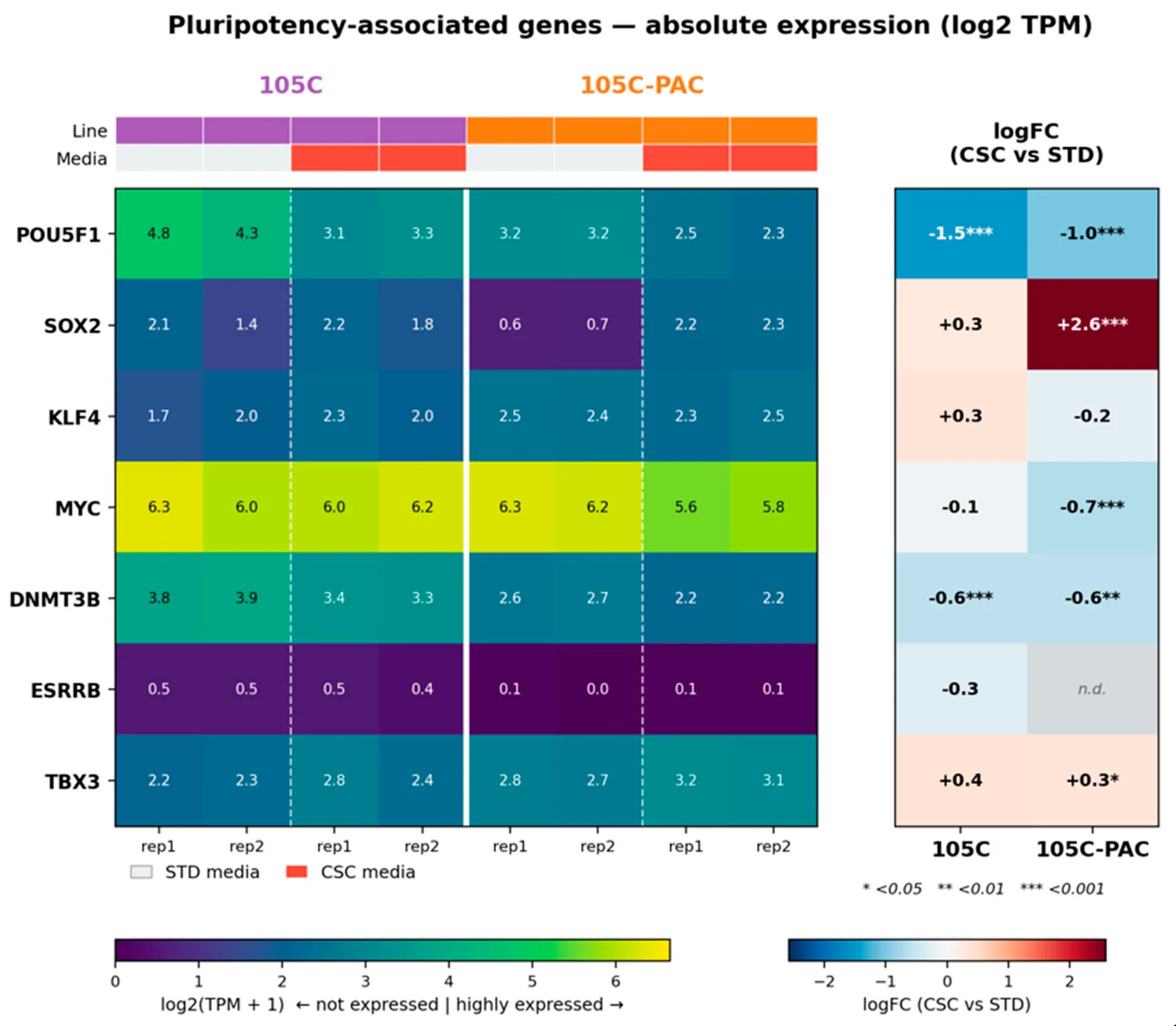
Core pluripotency transcription-factor expression in parental 105C and paclitaxel-resistant 105C-PAC spheroids. (**Left**) Absolute expression (log2(TPM + 1)) of canonical pluripotency transcription factors across the eight 105C-lineage samples (parental 105C and 105C-PAC, each in STD and CSC media, *n* = 2). Top annotation bars encode cell line (purple = 105C, orange = 105C-PAC) and medium (grey = STD, red = CSC). (**Right**) Per-line CSC-vs-STD log2 fold change for each gene from the matched limma-voom comparison (red = up in CSC, blue = down in CSC); numerals give the log2 fold change and asterisks the Benjamini-Hochberg-adjusted significance (* adj. *p* < 0.05, ** adj. *p* < 0.01, *** adj. *p* < 0.001); “n.d.” marks genes removed by the low-expression filter. *POU5F1* and *DNMT3B* are repressed by CSC media in both lines and SOX2 is induced by CSC media specifically in 105C-PAC, while the remaining factors change little.

Finally, because the resistant lines demonstrated enrichment of stem-associated markers under CSC culture conditions (Figure 7 and Figure 8), we tested whether resistance is accompanied by a gain in the core pluripotency programme by examining canonical pluripotency transcription factors in both lines (Figure 14). We found no coordinated induction. *POU5F1* (encoding OCT4) and *DNMT3B* were significantly repressed in CSC media in both lines, and *MYC* was repressed in CSC media in the resistant line, while *KLF4*, *ESRRB*, and *TBX3* changed little. The single exception was *SOX2*, which was strongly and specifically induced by CSC media in the resistant line (log2 fold change ≈ +2.6; adj. *p* < 0.001) but not in the parental 105C line, albeit from a low baseline expression level. Taken together with the proliferation-biased line signature, these data indicate that paclitaxel resistance in the 105C cell line is associated with a proliferative transcriptional state and a selective induction of *SOX2*, rather than a global amplification of the stem-cell-like programme elicited by CSC media.

### 3.8. Potential Markers of OCCC CSCs

Ultimately, while ALDH1A1, nor other members of the ALDH family, appear to serve as a marker of CSC for established human OCCC cell lines, several other potential markers and pathways were revealed to potentially identify or aid in the identification of cancer stem cells. To find potential markers of stemness in OCCC CSCs of established cell lines, the expression of several transcription factors implicated in cancer stemness were evaluated [80,81,82]. Transcription factors with significantly altered expression in CSC spheroids may have widespread effects on the transcriptome, potentially playing a key role in maintaining the stem-like phenotype. These transcription factors may also serve as biomarkers of CSC enrichment in OCCC and provide valuable targets for future studies investigating CSC populations.

**Figure 15:**
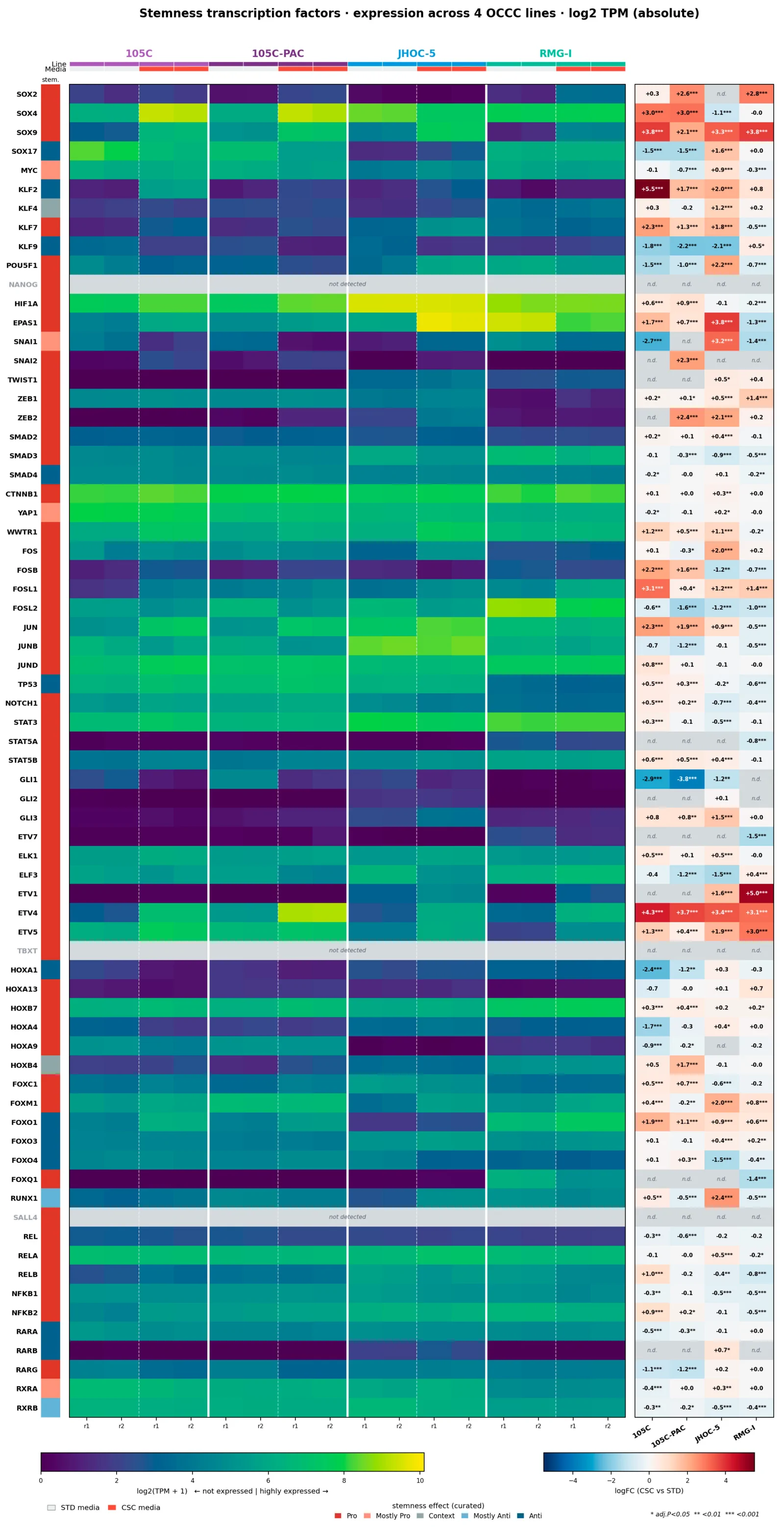
Expression of curated stemness transcription factors across the four OCCC cell line spheroids in CSC and standard media. A panel of 70 curated stemness-associated transcription factors, grouped by family, was profiled across all four OCCC lines (105C, the paclitaxel-resistant subline 105C-PAC, JHOC-5, and RMG-I) in CSC and standard (STD) media (*n* = 2 biological replicates per line and medium; 16 samples). (**Left**) Absolute expression, shown as log2(TPM + 1). Samples are ordered by cell line and then by medium, with solid white lines separating cell lines and dashed white lines separating STD from CSC within each line; replicates are labelled r1 and r2. Top annotation bars encode cell line (105C, purple; 105C-PAC, dark purple; JHOC-5, blue; RMG-I, teal) and medium (grey = STD, red = CSC). Three factors (NANOG, TBXT, and SALL4) were absent from the count matrix in all 16 samples and are rendered as grey “not detected” rows. (**Right**) Per-line CSC-versus-STD log2 fold change for each factor (red = up in CSC media, blue = down in CSC media). Numbers indicate log2 fold change and asterisks the Benjamini–Hochberg-adjusted significance (* adj. *p* < 0.05, ** adj. *p* < 0.01, *** adj. *p* < 0.001); grey “n.d.” cells mark factors removed by the low-expression filter in that line’s comparison.

The expression of several transcription factors implicated in cancer stemness were evaluated across all four cell lines. Of the 70 evaluated transcription factors, this analysis highlighted three as potential markers shared across all four cell lines: *SOX9*, *ETV4*, and *ETV5* (Figure 15) [80,81,82]. These markers demonstrated coordinated enrichment that was strong and specific to CSC media relative to STD media. This coordinated enrichment of *SOX9*, *ETV4*, and *ETV5* is consistent with their known transcriptional activity as SOX9 has been shown to influence *ETV4* and *ETV5* expression. Additionally, SOX9 and ETV5 have been demonstrated to work cooperatively to promote progenitor self-renewal programming [83]. Together, these markers represent a transcriptional programming favouring progenitor maintenance and lineage plasticity, maintaining cells in an undifferentiated or semidifferentiated state [81,84].

Several other markers were also highlighted within a subset of cell lines, offering additional markers that may be context- or cell-line-dependent. While not universally enriched across all cell lines, these markers still offer value to future studies assessing stemness in OCCC CSCs: *SOX2*, *SOX4*, *KLF7*, *EPAS1*, *ZEB2*, *FOSL1*, *JUN*, *ETV1*, and *FOXO1* (Figure 15). SOX2 and SOX4, have been known to induce pluripotency (SOX2) and inhibit differentiation (SOX4) [85,86]. KLF7 has been shown to maintain the reprogramming capabilities of KLF4 in inducing pluripotency in somatic cells [87]. *EPAS1* (HIF2A) has been shown to promote self-renewal, a stem-like phenotype, and support drug resistance though the activation of survival pathways [88,89]. ZEB2 is a key regulator of EMT, promoting the acquisition of mesenchymal traits such as migration and invasion [90]. FOSL1 and JUN are AP-1 transcription factors and, together as a transcription factor family, have been shown to promote EMT and stem cell programming [91,92,93]. Like ETV4 and ETV5, ETV1 works to maintain cell fate and lineage plasticity [94]. Finally, there is FOXO1, whose function is context-dependent but has shown to maintain stemness and self-renewal in pro-stem cell contexts [95,96].

While these candidate markers provide a foundation for defining the true molecular identity of OCCC CSCs, further studies are needed to validate their functional roles and clinical relevance. Further investigation into these transcriptional regulators will improve our understanding of OCCC CSC biology and may support the development of more effective strategies for the identification and therapeutic targeting of this cell population.

## 4. Discussion

ALDH1A1 is a widely established marker of CSCs in various cancer types and has been associated with stemness in OCCC [48,97,98,99,100,101,102,103]. Here, however, we show that ALDH1A1 plays an ambiguous role in the identification of OCCC CSCs across a diverse collection of human OCCC cell lines. Using 16 established human OCCC cell lines, we show that *ALDH1A1* mRNA expression is not significantly correlated with the expression of hallmark stemness factors (c-MYC, NANOG, SOX2) within a bulk adherent cell population. When relating adherent *ALDH1A1* mRNA expression to proliferative ability in CSCs spheroid culture, this ambiguity is further highlighted. One might hypothesize that lines with high ALDH1A1 expression and activity in adherent and STD media spheroid culture would have a growth advantage in stem-promoting conditions, as these cells could be considered as primed for demonstrating stem cell characteristics. However, using seven cell lines (KOC-7c, OVTOKO, 105C, TOV-21G, EFO-27, RMG-I, JHOC5), we show that ALDH1A1 expression and activity in adherent and STD media spheroid culture do not offer this advantage in CSC media. Lines with little to no expression of *ALDH1A1* demonstrated greater proliferative capacity in CSC media relative to STD media (EFO-27, RMG-I, JHOC-5) compared to the ALDH1A1 high-expressing lines (KOC-7c, OVTOKO).

Using the same seven cell lines, protein levels of ALDH1A1, NANOG, and SOX2 were assessed to determine if changes in proliferation coincide with changes in CSC marker expression in bulk adherent cells and day 3 and day 7 STD and CSC media spheroids. Strikingly, ALDH1A1 expression was completely ablated in CSC media in the EFO-27 and JHOC-5 cell line spheroids, yet was strongly expressed in STD media. In KOC-7c and OVTOKO, two of the highest expressing OCCC lines with high ALDH activity determined by flow cytometry, ALDH1A1 expression decreased in CSC media. In contrast, 105C, TOV-21G, and RMG-I, three lines with low expression and activity, showed relatively even ALDH1A1 expression in STD and CSC media. Alternatively, SOX2 and NANOG demonstrated CSC-specific expression in CSC media relative to STD media, as demonstrated by the 105C, RMG-I, and JHOC-5 cell lines.

Using chronic paclitaxel and AZD-8055 treatment we generated an AZD-8055-resistant 105C cell line, and paclitaxel-resistant OVTOKO, TOV-21G, and 105C cell lines. These lines demonstrated increased expression of stem associated marker expression relative to their parental counterparts, suggesting resistance-based enrichment of CSCs. While not statistically significant, both paclitaxel- and AZD-8055-resistant 105C cells demonstrated ALDH1A1 expression enrichment in CSC media.

This CSC-specific expression of ALDH1A1 introduces ambiguity regarding the role of ALDH1A1 in identifying OCCC CSCs. The difference in expression between the parental 105C cell line and resistant 105C (PAC and AZD) cell lines suggests that *ALDH1A1* expression may be more closely associated with therapy resistance and stress-induced adaptations than with baseline CSC sphere-forming capabilities and characteristics. Considering the similar growth patterns to parental 105C cells, paclitaxel- and AZD-8055 resistance may offer alternative mechanisms of stemness that may be more dependent on ALDH1A1, while the mechanism of stemness in parental 105C cells may be more ALDH1A1-independent.

Our RNA-seq analysis showed that 105C, RMG-I and JHOC-5 CSC spheroid cells displayed molecular traits consistent with established stem cells and well-characterized CSCs of other cancers, among their commonly differentially expressed genes, suggesting that these spheres are enriched with OCCC CSCs. GSEA across the three cell lines showed gene sets associated with proliferation (E2F targets, G2/M checkpoint, and mitotic spindle) to be the strong biological drivers of a proliferation response by spheroid cells in CSC media relative to spheroids generated and maintained in STD media. This response could be interpreted as an induction of a stem-like response in CSC spheroids, but this requires direct assessment using measures of stem cell properties such as the ELDA (Extreme Limiting Dilution Analysis) assay [104,105]. Importantly, when we compared our CSC media spheroid consensus gene expression signature from the RNA-seq to a collection of 20 curated stemness gene sets, we observed significant overlap with 10 of these gene sets from the literature. This further demonstrated that CSC media induced stem-like molecular programming in OCCC cell line spheroids. The CSC media-up-regulated gene set data highlighted enrichment in embryonic stem-cell signatures, transcriptional targets of NANOG and SOX2 from the Benporath gene set, embryonic stem-cell transcription-factor and iPSC signatures, and adult/mesenchymal stem-cell signatures (MSigDB) also listed in Supplementary Table S7.

Despite the RNA-seq data pointing to a stem-like transcriptome induced by CSC media in spheroids across all three cell lines, *ALDH1A*, or any other member of the ALDH family, did not show concordant enrichment in CSC spheroids. The exception was *ALDH18A1*, a Δ1-pyrroline-5-carboxylate synthase of proline biosynthesis, which was the only gene significantly concordantly up-regulated across all three lines. Thus, our data suggest that *ALDH1A1* is not induced by CSC media in OCCC cell line spheroids, implying that our model system does not exactly recapitulate CSC selection or that *ALDH1A1* is not an accurate marker of OCCC CSCs. Further studies using OCCC patient tumour cells applied to a large selection of potential stem cell markers by FLOW along with functional studies would provide foundational data elucidating the properties of human OCCC CSCs.

When comparing the effect of paclitaxel resistance on stemness in the 105C-PAC and 105C cells line, we found that resistant cell line spheroids maintained the molecular programming of the parental line. We looked at the expression of core pluripotency transcription factors and found transcriptional enrichment of SOX2 in the 105C-PAC cell line. Overall, paclitaxel resistance in the 105C cell line did not result in a global amplification of the stem-cell-like programme in CSC media described above. This suggested that for the 105C cell line, paclitaxel resistance did not drive a more stem-like state relative to the pre-resistant cell state. Our expectation was that drug resistance would induce a more robust conversion to a CSC-like phenotype relative to the parental cell line, exemplified by a coordinated induction of the core pluripotency transcription factors (*POU5F1*, *SOX2*, *NANOG*, *and KLF4*) together. Instead, this programme was not coordinately engaged: *SOX2* was the only pluripotency factor substantially induced (log2FC +2.6), with *TBX3* rising only marginally (+0.3), while *POU5F1* (OCT4), *MYC*, and *DNMT3B* were repressed in CSC media. Clearly, the 105C cell line did not acquire such characteristics based on our measures. An in vivo ELDA using immunocompromised mice may uncover a greater proportion of CSCs from the drug-resistant line when cultured as spheres in CSC media or even as a monolayer in STD media.

Two different studies, by Kuroda et al. and Wang et al., looked at ALDH1A1 in OCCC CSCs using limiting dilution xenotransplantation assays in immunocompromised mice, and came to different conclusions about ALDH1A1 as a singular marker of OCCC CSCs [98,99]. Both studies used ALDEFLUOR^TM^ FACS-sorted ALDH^high^ and ALDH^low^ cells injected at various limiting cell densities into immunocompromised mice using similar experimental methods. Kuroda et al. concluded that high ALDH activity alone marks OCCC CSCs based on experiments using the RMG-I cell line, with mice sacrificed seven weeks post-injection, whereas the other study reached the opposite conclusion using the TOV-21G cell line, with mice sacrificed six to nine weeks post-injection. Wang et al. noted that both ALDH^high^ and ALDH^low^ cells maintained the ability to produce heterogenous tumours in immunodeficient mice, albeit at differing efficiencies, indicating that ALDH^low^ cells still maintained some degree of tumorgenicity. ALDH ^low^ cells also demonstrated the ability to produce ALDH^high^ cells, furthering the stem-like capabilities of ALDH^low^ cells.

Comparing these two studies, high ALDH activity may play a cell-line- or context-dependent role in the identification of OCCC CSCs; however, neither study accounted for the promiscuity of ALDEFLUOR^TM^ FLOW cytometric assay. Both studies attributed high ALDH activity to ALDH1A1 specifically, yet the ALDEFLUOR^TM^ assay is known to detect the activity of several ALDH family members, with additional undetected members also implicated in promoting stemness in CSCs [106,107]. Given our result, it becomes more likely that the different conclusions reached by Kuroda et al. and Wang et al. could be due to attributing CSC activity of ALDH^high^ cells to ALDH1A1 alone, exacerbated by the use of a single OCCC cell line in both studies. It also provides a source to the ambiguity of ALDH1A1 as a marker of OCCC CSCs, as any ALDH family member could be responsible for the stem-like characteristics demonstrated by ALDH^high^ cells, in a manner that may be cell-line-dependent.

Our study took an enrichment approach, applying conditions to OCCC cell lines that reveal intrinsic CSC characteristics by culturing in CSC media as 3D spheroids. We assume this approach results in a heterogeneous population of stem-like cells that may mirror those found in patient tumours. Our approach was a general one based on assessing functional characteristics of spheroids formed in CSC enriching media followed by an unbiased approach using bulk RNA-seq, therein eliminating biomarker bias allowing the biology of these spheroids to reveal markers that may be generalizable.

Identifying the changes in the mRNA expression of established CSC markers, and the transcriptional targets of the transcription factors and pathways implicated in stemness, provides a foundation for identifying protein markers that can be used in isolating CSCs from bulk cell tumour populations. The transcription factors we identified as up-regulated in CSC media spheroids may be relevant to the analyses of OCCC tumours formed in vivo using a limiting dilution xenograft model in immunocompromised mice. Our RNA-seq data clearly show that CSC-media generation of OCCC cell line spheroids enriches for a CSC-like transcriptome, thus providing a way to further investigate gene expression patterns that would define an OCCC CSC. Examining 15 different OCCC cell lines may assist future studies of OCCC CSC plasticity and how this property might be disrupted from a therapeutic perspective.

Our approach has provided a foundation for future studies investigating the transcriptional, epigenetic, and biological features that distinguish OCCC CSCs from bulk tumour cells. Applying our data regarding transcription factors which may mark OCCC CSCs to primary human tumour specimens may facilitate new ways of isolating and studying OCCC CSC properties. The goal of such studies would be to identify biological vulnerabilities as therapeutic opportunities against OCCC tumour biology.

## Figures and Tables

**Table 1:** Primers used for PCR and RT-qPCR.

Gene	Primer Sequence		Sequence Source
***hALDH1A1***	**F**	TGTTAGCTGATGCCGACTTG	[50]
	**R**	TTCTTAGCCCGCTCAACACT	
***hSOX2***	**F**	TACAGCATGTCCTACTCGCAG	PrimerBank ID: 325651854c3 [51,52,53]
	**R**	GAGGAAGAGGTAACCACAGGG	
***hNANOG***	**F**	TTTGTGGGCCTGAAGAAAACT	PrimerBank ID: 153945815c1 [51,52,53]
	**R**	AGGGCTGTCCTGAATAAGCAG	
***hKLF4***	**F**	AAGCACTGGGGGAAGTCGCTTC	[54]
	**R**	ATCTTTCTCCACGTTCGCGTCTG	
***hMYC***	**F**	GCTGCTTAGACGCTGGATTT	[55]
	**R**	CACCGAGTCGTAGTCGAGGT	
***hPOU5F1***	**F**	CTGGAGAAGGAGAAGCTGGA	[56]
	**R**	CAAATTGCTCGAGTTCTTTCTG	
***hGAPDH***	**F**	CATGAGAAGTATGACAACAGCCT	[57]
	**R**	AGTCCTTCCACGATACCAAAGT	

**Table 2:** Antibodies used for immunoblotting.

Antibody	Dilution	Manufacturer	Catalogue Number
ALDH1A1	1:1000	Cell Signalling Technology (Danvers, MA, USA)	#54135
SOX2	1:1000	Cell Signalling Technology	#3579
NANOG	1:2000	Cell Signalling Technology	#4903
Vinculin	1:10,000	Sigma-Aldrich	#V9264
Caspase 3	1:1000	Cell Signalling Technology	#9662
Cleaved Caspase 3 (Asp175)	1:1000	Cell Signalling Technology	#9661
HRP-Rabbit	1:10,000	Cytiva (Malborough, MA, USA)	#NA934
HRP-Mouse	1:10,000	Cytiva	#NA931

**Table 3:** Maintenance concentrations (nM) of paclitaxel and AZD-8055 for drug-resistant OCCC cell lines.

Cell Line	Paclitaxel (nM)	AZD-8055 (nM)
OVTOKO-PAC	532.09	-
TOV-21G-PAC	8.186	-
105C-PAC	5.9682	-
105C-AZD	-	154.176

**Table 4:** IC50 (nM) values of resistant cell lines.

Cell Line	Parental IC50 (nM)	Resistant IC50 (nM)	Fold Change
OVTOKO-PAC	124.9	1388	11.11
TOV-21G-PAC	4.326	33.91	7.839
105C-PAC	3.036	9.780	3.221
105C-AZD	34.31	746.3	21.75

## Data Availability

Scripts are available upon request. The RNA-Seq data presented in this study are available in the NCBI Gene Expression Omnibus (GEO) database (https://www.ncbi.nlm.nih.gov/geo/ accessed on 1 June 2026) under GEO accession number GSE338969.

## Supplementary Materials

The following supporting information can be downloaded at: https://www.mdpi.com/article/10.3390/cells15171509/s1, Figure S1: ALDH1A1 Western blot quantification; Figure S2: OCCC cell line spheroid morphology in STD and CSC media; Figure S3: Quantification graphs showing changes in CSC marker protein levels from cells cultured in STD and CSC media; Figure S4: Quantification graphs of changes in CSC marker protein levels in drug-resistant cell lines cultured in STD and CSC media; Figure S5 ALDH gene family expression in 105C cell lines; Table S1: OCCC cell line usage in this study; Table S2: Significance values of Spearman correlation analyses for the expression of Yamanaka Factors and ALDH1A1 in OCCC cell lines; Table S3: OCCC cell line summary data table; Table S4: CSC spheroid growth observations for 19 cell lines used in this study; Table S5: List of concordant gene expression changes in spheroids generated by culturing in CSC or STD media across the 105C, JHOC-5 and RMG-I cell lines; Table S6: Overlap of the down-regulated gene expression signature induced by CSC media with established curated stemness gene sets; Table S7: Overlap of the up-regulated gene expression signature induced by CSC media with established curated stemness gene sets; Table S8: Assessment of the genes in Table S5 using Enrichr identified the single-cell gene expression database, PanglaoDB, which identified Pluripotent Stem Cells signature as closely matching with the up-regulated gene set induced in CSC media spheroids; Table S9: List of all differentially expressed genes in the 105C-PAC cell line spheroids comparing CSC media to STD media.

## Abbreviations

The following abbreviations are used in this manuscript:

ALDH	aldehyde dehydrogenase
ANOVA	analysis of variance
ATCC	American Type Culture Collection
BAAA	BODIPY-aminoacetaldehyde
bFGF	basic fibroblast growth factor
BME	beta-mercaptoethanol
BSA	bovine serum albumin
CPM	counts per million
CSC	cancer stem cell
DEAB	4-diethylaminobenzaldehyde
DMEM/F12	Dulbecco’s Modified Eagle Medium/Ham’s F12
EOC	epithelial ovarian cancer
FACS	fluorescence-activated cell sorting
FBS	fetal bovine serum
FDR	false discovery rate
GSEA	gene set enrichment analysis
hEGF	human epidermal growth factor
HEPES	4-(2-hydroxyethyl)-1-piperazineethanesulfonic acid
HGSOC	high-grade serous ovarian carcinoma
IC50	half-maximal inhibitory concentration
ITSS	insulin-transferrin-sodium selenite supplement
ML	monolayer
NES	normalized enrichment score
OCCC	ovarian clear cell carcinoma
PAC	paclitaxel (suffix denoting paclitaxel-resistant sublines)
PVDF	polyvinylidene fluoride
SDS	sodium dodecyl sulfate
SPH	spheroid
STD	standard (media)
STR	short tandem repeat
TCAG	The Centre for Applied Genomics
TMM	trimmed mean of M-values
TPM	transcripts per million
ULA	ultra-low attachment
