# Supplementary material for: Exploring the Utility of ALDH1 as a Marker for the Cancer Stem Cell Population in OCCC Cell Lines
Source: Cells. 2026 Aug 22;15(17):1509. doi: 10.3390/cells15171509 (PMC13565638; doi:10.3390/cells15171509)
Supplement: Supplementary file 1 [file cells-15-01509-s001.zip › Figure S1. Spheroid morphology in STD and CSC media.pdf]

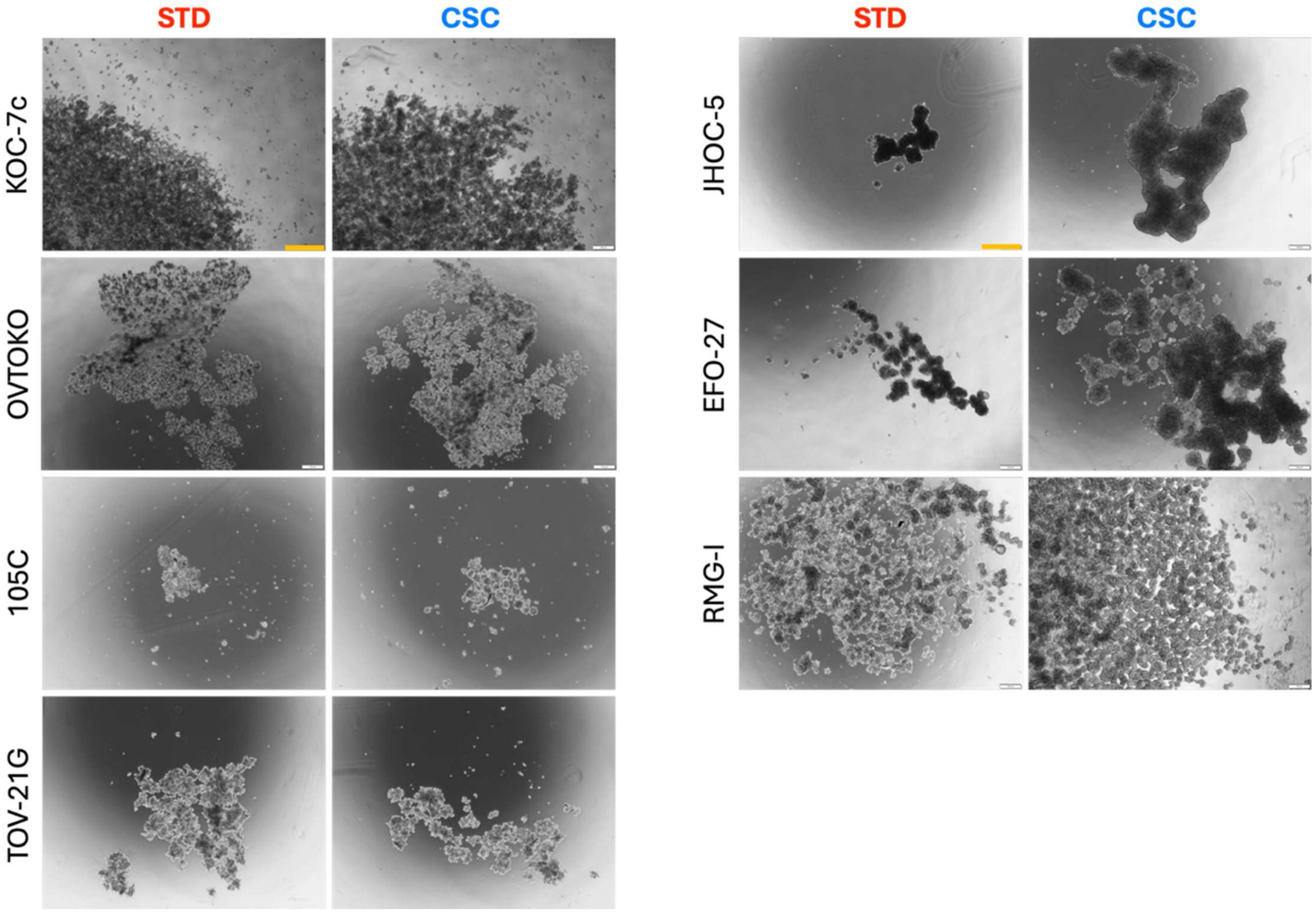

Figure S1. Spheroid morphology in STD and CSC media. Cells were seeded at a density of 1.0×10^4 cells/well in a 24-well ULA plate for both STD and CSC and imaged on day 7. Yellow bar represents 500 μM.
