# Supplementary material for: Exploring the Utility of ALDH1 as a Marker for the Cancer Stem Cell Population in OCCC Cell Lines
Source: Cells. 2026 Aug 22;15(17):1509. doi: 10.3390/cells15171509 (PMC13565638; doi:10.3390/cells15171509)
Supplement: Supplementary file 1 [file cells-15-01509-s001.zip › Figure S3. Protein quantification of changes in protein expression of CSC.pdf]

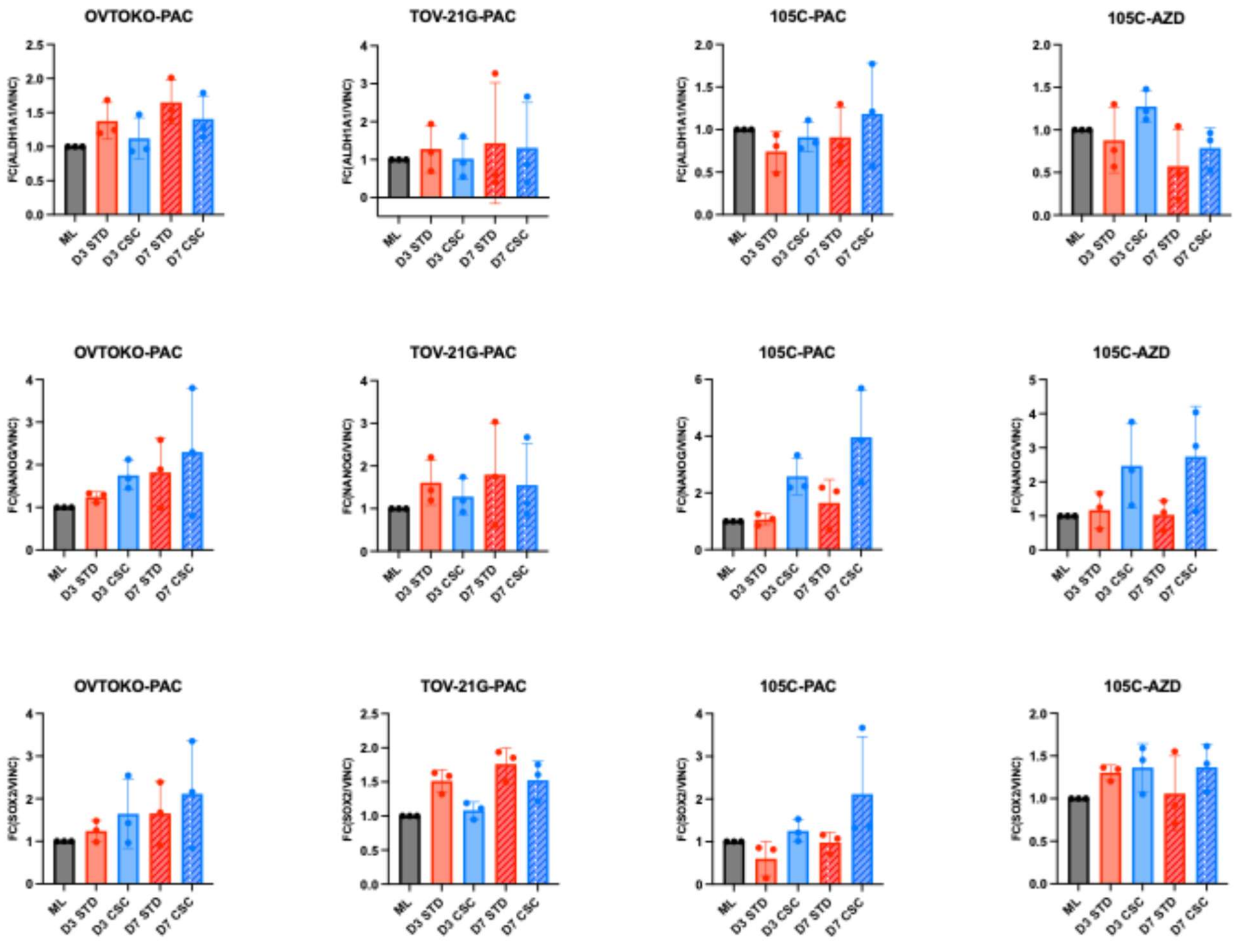

Figure S3. Protein quantification of changes in protein expression of CSC markers in adherent cells, day 3 and day 7 resistant cell line spheroids cultured in STD and CSC media. Cells were seeded in 6-well ULA plates at a cell density of 1-2 × 10^5 cells/well in STD or CSC media. Adherent cells were seeded at 0.5-1 × 10^6 cells per 10 cm dish in STD media. Whole cell protein lysates were collected on day 3 for adherent cells and spheroids, and again on day 7 for spheroids.
