# Supplementary material for: Exploring the Utility of ALDH1 as a Marker for the Cancer Stem Cell Population in OCCC Cell Lines
Source: Cells. 2026 Aug 22;15(17):1509. doi: 10.3390/cells15171509 (PMC13565638; doi:10.3390/cells15171509)
Supplement: Supplementary file 1 [file cells-15-01509-s001.zip › Table S1. Significance values of Spearman correlation analyses.pdf]

Table S1. Significance values of Spearman correlation analyses for the expression of Yamanaka Factors and ALDH1A1 in OCCC cell lines. Correlations where $p<0.05$ are highlighted in grey.

	ALDH1A1	NANOG	SOX2	KLF4	c-MYC
**ALDH1A1**	-	0.430	0.527	0.221	0.961
**NANOG**	0.430	-	8.381E-05	0.070	0.014
**SOX2**	0.527	8.381E-05	-	0.021	0.022
**KLF4**	0.221	0.070	0.021	-	0.104
**c-MYC**	0.961	0.014	0.022	0.104	-
