# Supplementary material for: Exploring the Utility of ALDH1 as a Marker for the Cancer Stem Cell Population in OCCC Cell Lines
Source: Cells. 2026 Aug 22;15(17):1509. doi: 10.3390/cells15171509 (PMC13565638; doi:10.3390/cells15171509)
Supplement: Supplementary file 1 [file cells-15-01509-s001.zip › Table S2.CSC spheroid growth observations for 22 cell lines used in this study.pdf]

Table S2. CSC spheroid growth observations for 22 cell lines used in this study.
OCCC denotes ovarian clear cell carcinoma, ENDO (SV40) denotes SV40-induced immortalized endometriosis, HGSOC denotes high-grade serous ovarian carcinoma. *EFO27 is officially considered to be a mucinous ovarian cancer cell line; however, here it is classified as OCCC as its mutational profile is consistent with OCCC tumours.

Cell line	Histotype	STD Spheroid Growth	CSC Spheroid Growth and Formation Screen				CSC Spheroid Growth
			250 cells/well	500 cells/well	1000 cells/well	1500 cells/well	
**105C**	OCCC	No	No spheroids	No spheroids	No spheroids	No spheroids	Yes
**ES-2**	OCCC	Yes	No spheroids	Numerous Spheroids at D10	Numerous Spheroids at D5	Numerous Spheroids at D5	Yes
**JHOC-5**	OCCC	Yes	No spheroids	No spheroids	Numerous Spheroids at D5	Numerous Spheroids at D5	Yes
**KOC-7c**	OCCC	Yes	Numerous Spheroids at D10	Abundant Spheroids at D5	Abundant Spheroids at D5	Abundant Spheroids at D5	Yes
**OVTOKO**	OCCC	Yes	No spheroids	Few spheroids at D10	Few spheroids at D5	Few spheroids at D5	Yes
**SMOV2**	OCCC	No	Few spheroids at D10	Few spheroids at D5	Few spheroids at D5	Few spheroids at D5	Yes
**TOV-21G**	OCCC	Yes	No spheroids	Few spheroids at D5	Few spheroids at D5	Few spheroids at D10 enlarged	Yes
**TU-OC-1**	OCCC	No	No spheroids	Few spheroids at D5 & enlarged at D10	Few spheroids at D5 & enlarged at D10	Few spheroids at D5 & enlarged at D10	Yes
**59M**	OCCC	No	No spheroids	No spheroids	No spheroids	No spheroids	No

**OV207**	OCCC	No	2 spheroids	~10 small spheroids	Many more spheroids	Many more spheroids	Yes
**OVISE**	OCCC	No	Very few small spheroids	Lots of small spheroids	Lots of small spheroids	Lots of small spheroids	Yes
**OVMANA**	OCCC	No	No spheroids	Lots of small spheroids	Lots of small spheroids	Lots of small spheroids	No
**OVSAYO**	OCCC	No	No spheroids	Several tiny spheroids or cell debris	Several tiny spheroids or cell debris	Several tiny spheroids or cell debris	No
**RMG-I**	OCCC	Yes	Lots of large clusters	Lots of large clusters	Lots of large clusters	Lots of large clusters	Yes
**RMG-II**	OCCC	No	Tiny clusters or cell debris	Tiny clusters or cell debris	No spheroids	No spheroids	No
**RMG-V**	OCCC	No	Several small spheroids or debris	Several small spheroids or debris	Several small spheroids or debris	Several small spheroids or debris	Yes
**12Z**	ENDO (SV40)	Yes	No spheres	No spheres	Few small spheres day 5, appear to be dying by day 10	Few small spheres on day 5, larger but appear to be dying by day 10	Yes
**EFO-21**	HGSOC	No	Very few and very small spheres by day 5, fewer small spheres by day 10	Very few and very small spheres by day 5, fewer small spheres by day 10	Many small spheres by day 5, larger by day 10	Many small spheres by day 5, larger by day 10	Yes
**EFO-27**	OCCC*	No	Few spheres on day 5, few	Few spheres on day 5, few	Few spheres on day 5, few	Few spheres on day 5, few	Yes

		larger spheres by day 10	larger spheres by day 10	larger spheres by day 10	larger spheres by day 10	
