# Supplementary material for: Exploring the Utility of ALDH1 as a Marker for the Cancer Stem Cell Population in OCCC Cell Lines
Source: Cells. 2026 Aug 22;15(17):1509. doi: 10.3390/cells15171509 (PMC13565638; doi:10.3390/cells15171509)
Supplement: Supplementary file 1 [file cells-15-01509-s001.zip › ✅Supplementary Figure S4 Protein quantification of CSC markers in drug resistant cell lines.pdf]

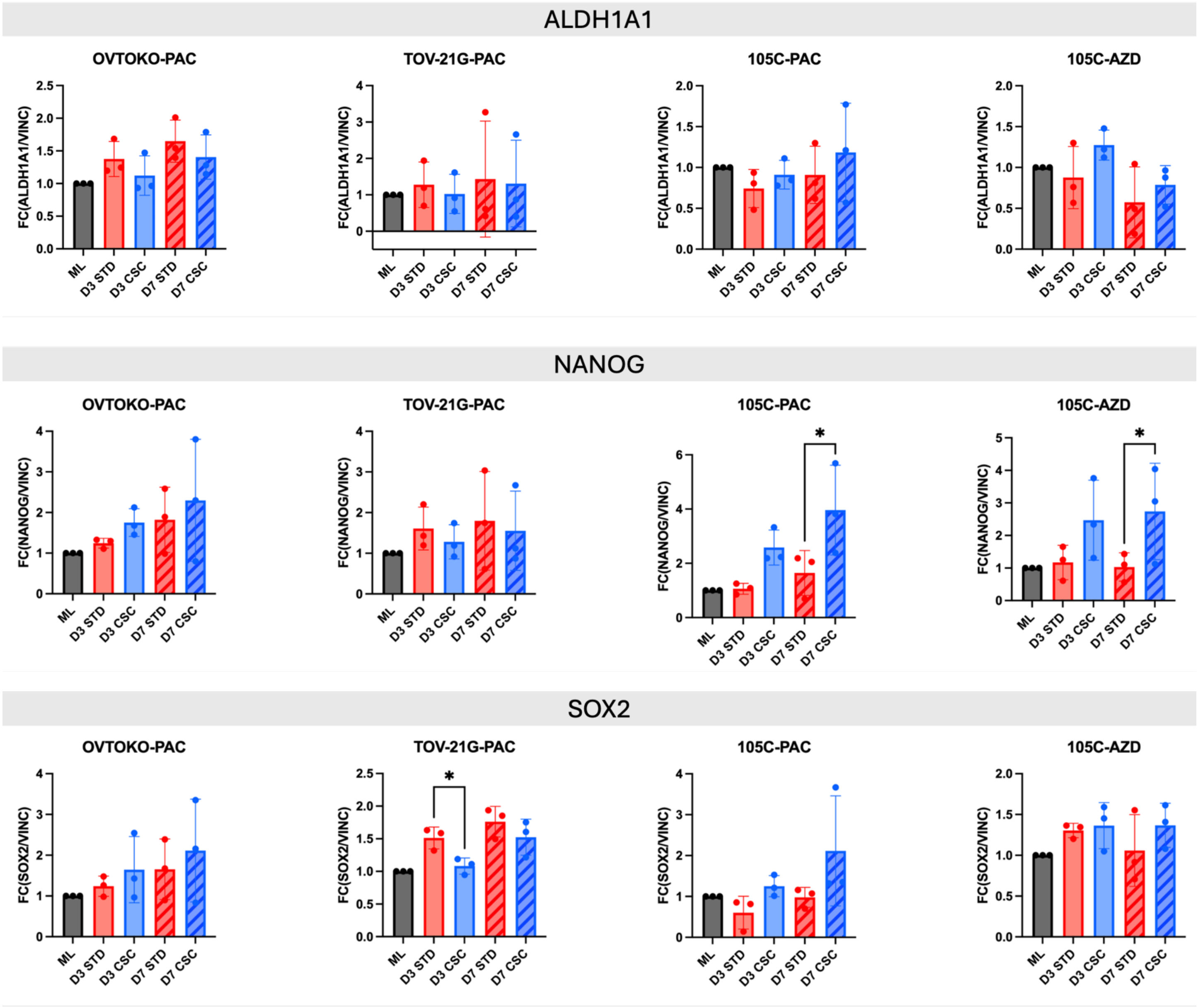

Supplementary Figure S4. Protein quantification of changes in CSC marker levels in adherent cells (ML), and day 3 and day 7 drug-resistant cell line spheroids cultured in STD and CSC media. Cells were seeded in 6-well ULA plates at a cell density of 1-2 × 10^5 cells/well in STD or CSC media. Adherent cells were seeded at 0.5-1 × 10^6 cells per 10 cm dish in STD media. Whole cell protein lysates were collected on day 3 for adherent cells and spheroids, and again on day 7 for spheroids. PAC: Paclitaxel; AZD: AZD-8055 mTOR inhibitor. Data represents ML normalized protein quantification from 3 independent biological replicates; two-way ANOVA with simple effects post hoc comparisons (*$p<0.05$).
