# Supplementary material for: Exploring the Utility of ALDH1 as a Marker for the Cancer Stem Cell Population in OCCC Cell Lines
Source: Cells. 2026 Aug 22;15(17):1509. doi: 10.3390/cells15171509 (PMC13565638; doi:10.3390/cells15171509)
Supplement: Supplementary file 1 [file cells-15-01509-s001.zip › ✅Supplementary Figure S5 ALDH gene family expression in 105C cell lines.pdf]

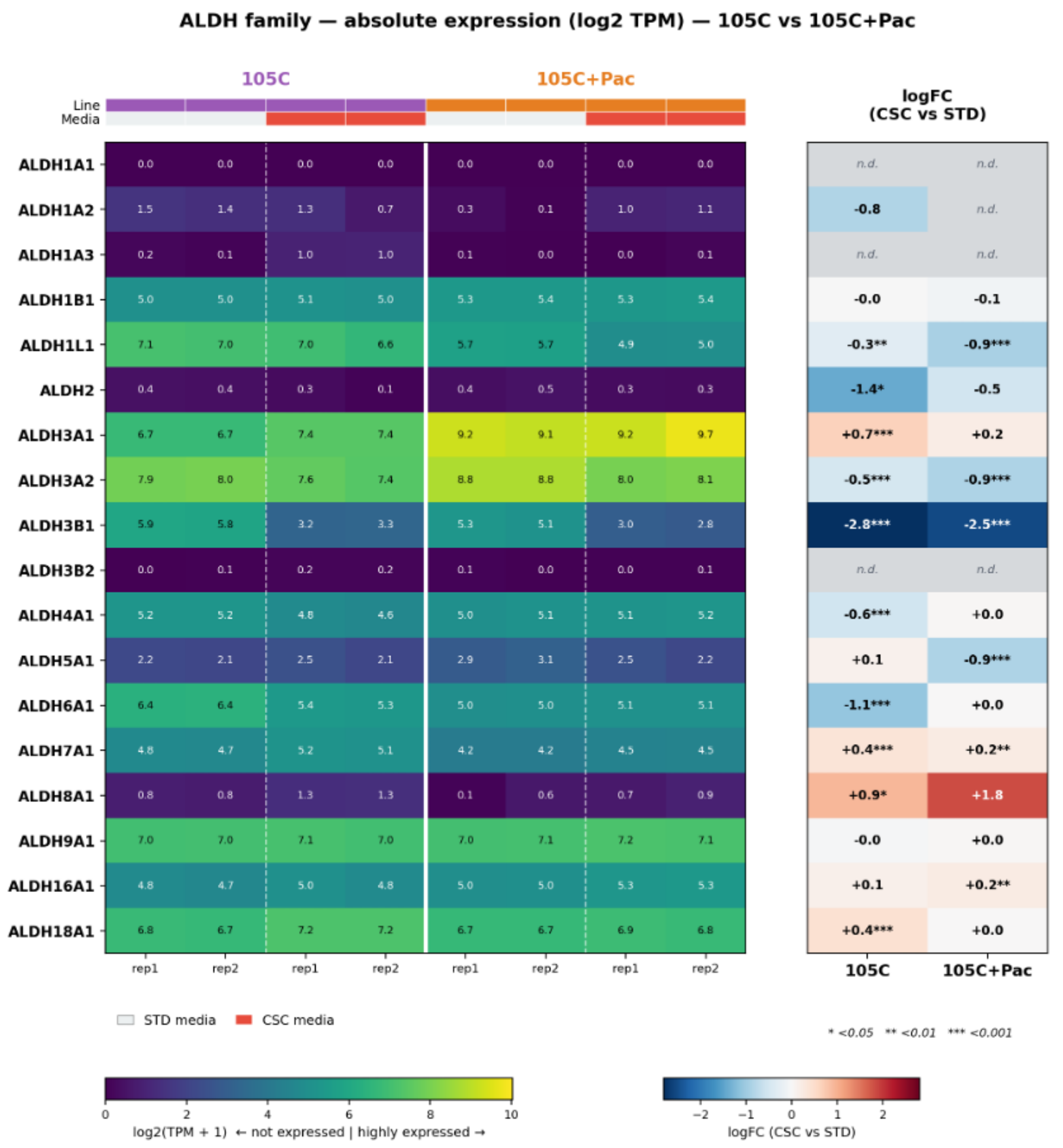

Supplementary Figure S5. Expression of ALDH gene family members in the 105C parental cell line and the paclitaxel-resistant 105C spheroids cultured in STD and CSC media. (Left panel) Absolute expression of 18 protein-coding ALDH family members, displayed as log2(TPM + 1) across all 8 RNA-seq samples (n = 2 per cell-line × media combination). TPM values were derived from the limma-voom-normalised log2 CPM matrix using Ensembl canonical transcript lengths. Annotation bars at top encode cell-line identity (purple = 105C, orange = 105C-PAC) and culture media (light grey = standard / STD, red = cancer stem cell / CSC). (Right panel) Per-cell-line CSC-vs-STD log2 fold change for each ALDH gene from the corresponding limma-voom DE comparison. Significance asterisks: * adj.P < 0.05, ** adj.P < 0.01, *** adj.P < 0.001 (Benjamini–Hochberg-corrected within each per-line DE comparison).

B. Gebreyes et al. 2026; Exploring the utility of ALDH1 as a marker for the cancer stem cell population in OCCC cell lines
